# Bcl-2 inhibition combined with PPARα activation synergistically targets leukemic stem cell-like cells in acute myeloid leukemia

**DOI:** 10.1038/s41419-023-06075-6

**Published:** 2023-08-29

**Authors:** Chendi Xie, Hui Zhou, Dongmei Qin, Huijian Zheng, Yuanfang Tang, Wenjuan Li, Jie Zhou, Long Liu, Xinxin Yu, Hongpeng Duan, Yong Zhou, Zhifeng Li, Zhihong Fang, Yiming Luo, Bing Z. Carter, Bing Xu, Jie Zha

**Affiliations:** 1 Department of Hematology, the First Affiliated Hospital of Xiamen University and Institute of Hematology, School of Medicine, Xiamen University, Xiamen, China; 2 Key Laboratory of Xiamen for Diagnosis and Treatment of Hematological Malignancy, Xiamen, China; 3 State Key Laboratory of Cellular Stress Biology, Innovation Center for Cell Biology, School of Life Sciences, Xiamen University, Xiamen, China; 4 School of Clinical Medicine, Fujian Medical University, Fuzhou, Fujian, China; 5 Section of Molecular Hematology and Therapy, Department of Leukemia, the University of Texas MD Anderson Cancer Center, Houston, Texas USA

**Keywords:** Acute myeloid leukaemia, Apoptosis

## Abstract

Persistence of leukemic stem cells (LSCs) is one of the determining factors to acute myeloid leukemia (AML) treatment failure and responsible for the poor prognosis of the disease. Hence, novel therapeutic strategies that target LSCs are crucial for treatment success. We investigated if targeting Bcl-2 and peroxisome proliferator activated receptor α (PPARα), two distinct cell survival regulating mechanisms could eliminate LSCs. This study demonstrate that the Bcl-2 inhibitor venetoclax combined with the PPARα agonist chiglitazar resulted in synergistic killing of LSC-like cell lines and CD34^+^ primary AML cells while sparing their normal counterparts. Furthermore, the combination regimen significantly suppressed AML progression in patient-derived xenograft (PDX) mouse models. Mechanistically, chiglitazar-mediated PPARα activation inhibited the transcriptional activity of the *PIK3AP1* gene promoter and down-regulated the PI3K/Akt signaling pathway and anti-apoptotic Bcl-2 proteins, leading to cell proliferation inhibition and apoptosis induction, which was synergized with venetoclax. These findings suggest that combinatorial Bcl-2 inhibition and PPARα activation selectively eliminates AML cells in vivo and vitro, representing an effective therapy for patients with relapsed and refractory AML.

## Introduction

Acute myeloid leukemia (AML) is the most common subtype of adult leukemia. Currently, the five-year survival rate is only approximately 30% and improved therapeutic approaches are needed [1]. Leukemia stem cells (LSCs), a subpopulation of leukemic cells are characterized with CD34^+^/CD34^+^CD38^-^ phenotypes that can self-renew and initiate leukemia [2]. Their quiescent nature and unique protective mechanisms, such as resistance to apoptosis, make them less sensitive to chemotherapy, which is also the root cause of AML recurrence [3–6].

Bcl-2 is an anti-apoptotic protein that protects leukemia cells and LSCs from therapeutic agent-induced apoptosis. Venetoclax (ABT-199), the highly selective Bcl-2 inhibitor, has entered clinical applications for treating patients with hematological malignancies [7]. However, the overall remission rate (ORR) of venetoclax monotherapy for high-risk relapsed refractory (R/R) AML is only 19%, and the overall survival (OS) of patients is only 4.7 months [8, 9]. Combination of venetoclax with hypomethylating agents has greatly improved response rates, but OS is still only 14.7 months [10, 11]. Multiple mechanisms including dependency on alternative anti-apoptotic proteins Bcl-xL or Mcl-1 contribute to venetoclax resistance [12], supporting that co-targeting more than one cell survival mechanisms is needed to improve the efficacy of venetoclax in AML [13].

PPAR is a transcription factor. PPAR-retinoid X receptor (RXR) heterodimer binds with peroxisome proliferators responsive elements (PPREs) in the promoter region of target genes to regulate the transduction of downstream genes [14–16]. Chiglitazar, an agent currently used for treating type II diabetes has been identifies as a PPAR agonist [17], significantly induces apoptosis by enhancing the expression of PPARα in HepG2 cells [18] and triggers apoptosis in tumor cells in glioma [19] and mantle cell lymphoma (MCL) [20]. Additionally, the activation of PPARα can promote the degradation of HIF-1α under hypoxic conditions, thereby down-regulating the target gene *CYP3A4* in hepatoma cells [21] and *VEGF* in MCF-7 human breast cancer cells and A2780 ovarian cancer cells [22]. These studies indicate that chiglitazar could serve as a low-toxicity adjuvant drug for treating tumors.

Here, we demonstrate that combined inhibition of Bcl-2 by venetoclax and activation of PPARα by chiglitazar has synergistic anti-leukemia activity against LSC-like cell lines (KG-1α and Kasumi-1) and CD34^+^ primary AML cells while sparing normal cells in vitro. Besides, the combination of venetoclax and chiglitazar significantly inhibited AML progression in PDX mouse models driven by CD34^+^ primary AML cells. Mechanistically, the combination regimen targets multiple members of Bcl-2 family proteins: venetoclax inhibits Bcl-2 and chiglitazar regulates the expression of Bcl-2 family proteins by suppression the PI3K/Akt signaling pathway mediated through PPARα activation.

## Results

### Venetoclax combined with chiglitazar synergistically decreases the viability of LSC-like cells in vitro

**Fig. 1:**
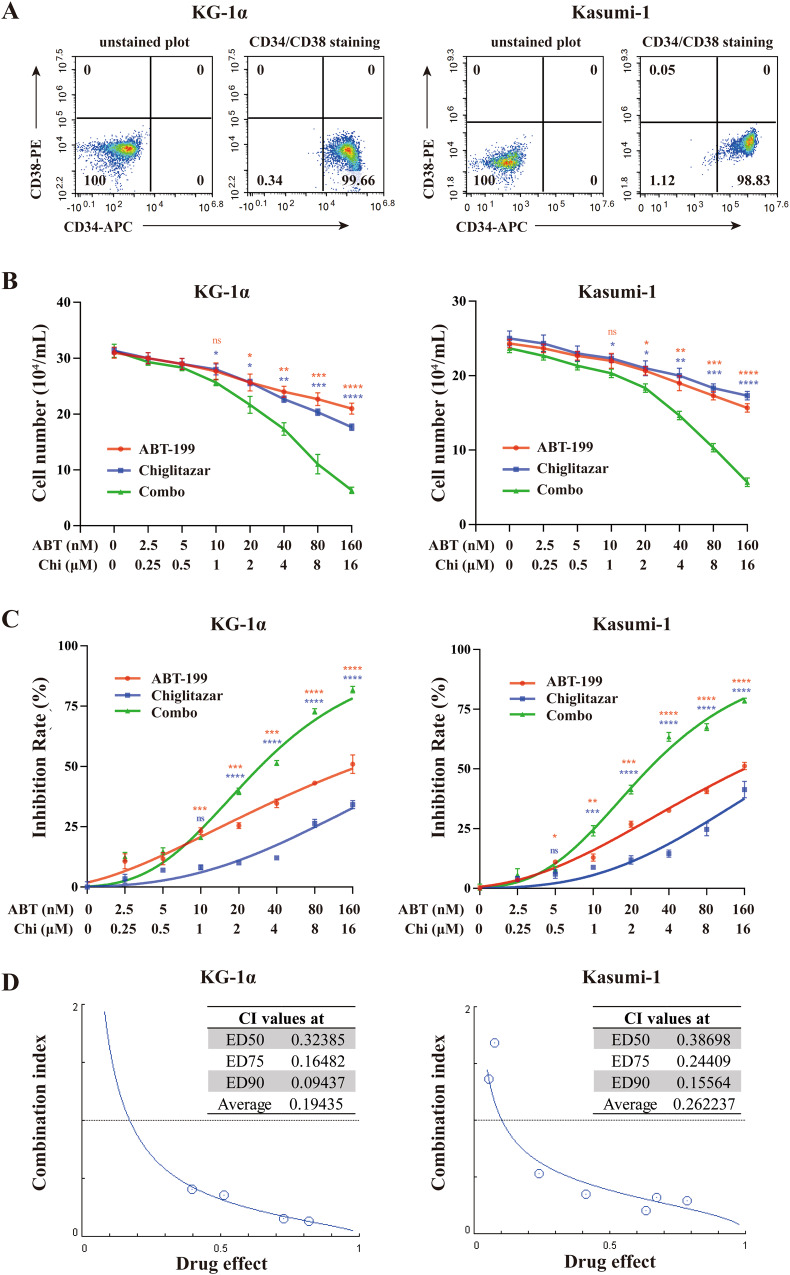
Venetoclax combined with chiglitazar synergistically inhibited the proliferation of LSC-like cells in vitro. **A** Representative FACS analysis for determining the percentage of CD34^+^CD38^−^ cells enriched from KG-1α and Kasumi-1 cells. **B**, **C** Living cells of KG-1α and Kasumi-1 were counted with trypan blue staining **B** and a CCK-8 assay was used to detect the anti-proliferative ability **C**. **D** Combination index (CI) plots showing venetoclax/chiglitazar combinatorial treatment in KG-1α and Kasumi-1 cells. Values represent mean ± SD for three replicates. *P < 0.05, **P < 0.01, ***P < 0.001 and ****P < 0.0001.

To document the phenotypic features of KG-1α and Kasumi-1 cell lines, flow cytometric analyses were performed to characterize the LSC-like CD34^+^CD38^-^ population (Fig. 1A) and examine the expression of multiple LSC-relevant markers, including CD123, CD96, CD25, CD44, CD32, and CD47 (Supplemental Fig. S1). Thus, these CD34^+^-enriched KG-1α and Kasumi-1 cells were used for further analyses in this study.

To evaluate the effect of venetoclax combined with chiglitazar on the viability of LSC-like cells, KG-1α and Kasumi-1 cells were cultured and treated with designated drugs. Trypan blue staining (Fig. 1B) revealed that venetoclax or chiglitazar decreased living cell numbers in a dose-dependent manner and the combination regimen further decreased cell viability, which was further confirmed by CCK-8 assays (Fig. 1C). Venetoclax and chiglitazar combination was highly synergistic in reducing the viability of KG-1α and Kasumi-1 cells (Fig. 1D).

### Venetoclax combined with chiglitazar synergistically induces LSC-like cell apoptosis in vitro

**Fig. 2:**
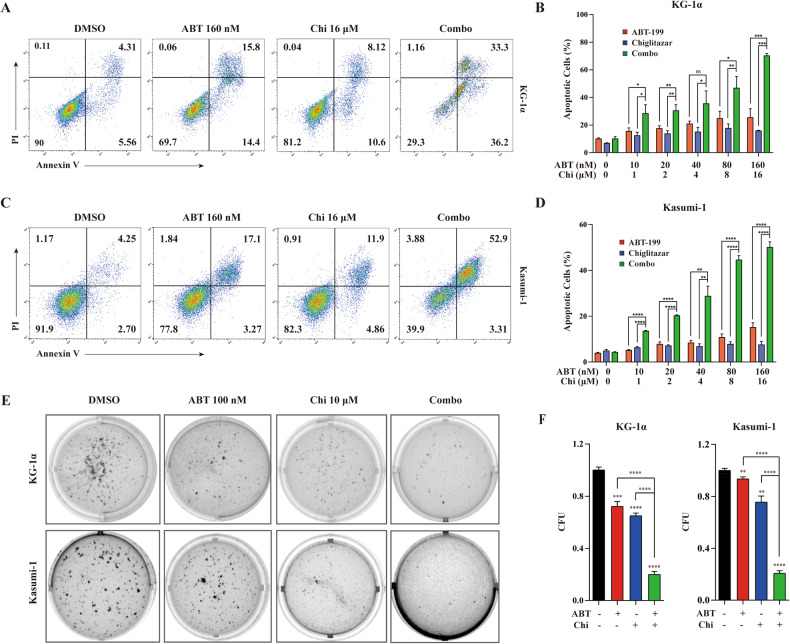
Venetoclax combined with chiglitazar synergistically induced the apoptosis and inhibited clone formation of LSC-like cells in vitro. **A**, **B** Representative flow cytometric data for Annexin V/PI staining of KG-1α **A** and percentages of apoptotic cells **B** were determined. **C**, **D** Representative flow cytometric data **C** and percentages of apoptotic **D** of Kasumi-1. **E**, **F** Clonogenic capacity identification of KG-1α and Kasumi-1 cells was performed by determining the percentage of CFUs. Values represent mean ± SD for three replicates. *P < 0.05, **P < 0.01, ***P < 0.001 and ****P < 0.0001.

Since PPARα activation can induce apoptosis in a variety of tumor cells [18–21] and venetoclax inhibits the anti-apoptotic protein Bcl-2 [23], the apoptosis of KG-1α cells was assessed by flow cytometry using Annexin V/PI staining (Supplemental Fig. S2A). KG-1α (Fig. 2A, B) and Kasumi-1 cells (Fig. 2C, D) were treated with designated drugs for 24 h. Each agent alone induced apoptosis with a dose-dependent manner, which was significantly enhanced when the two drugs were combined. The flow cytometry data and CI value results are shown in Supplemental Fig. S2. These results showed that venetoclax combined with chiglitazar synergistically induced apoptosis in KG-1α and Kasumi-1 cells in vitro.

### Venetoclax combined with chiglitazar synergistically inhibits clone formation in LSC-like cells

A soft agar clone formation experiment was used to assess the effect of venetoclax/chiglitazar on the tumor-forming ability of KG-1α and Kasumi-1 cells in vitro. After 24-h pretreatment with venetoclax (100 nM) or chiglitazar (10 μM) alone moderately inhibited colony formation of KG-1α and Kasumi-1 cells, while combination therapy had a significant effect (Fig. 2E, F; *P* < 0.0001 for each case). These results indicate that venetoclax combined with chiglitazar can significantly inhibit the colony forming ability of KG-1α and Kasumi-1 cells in vitro.

### Venetoclax combined with chiglitazar effectively down-regulates anti-apoptotic proteins

**Fig. 3:**
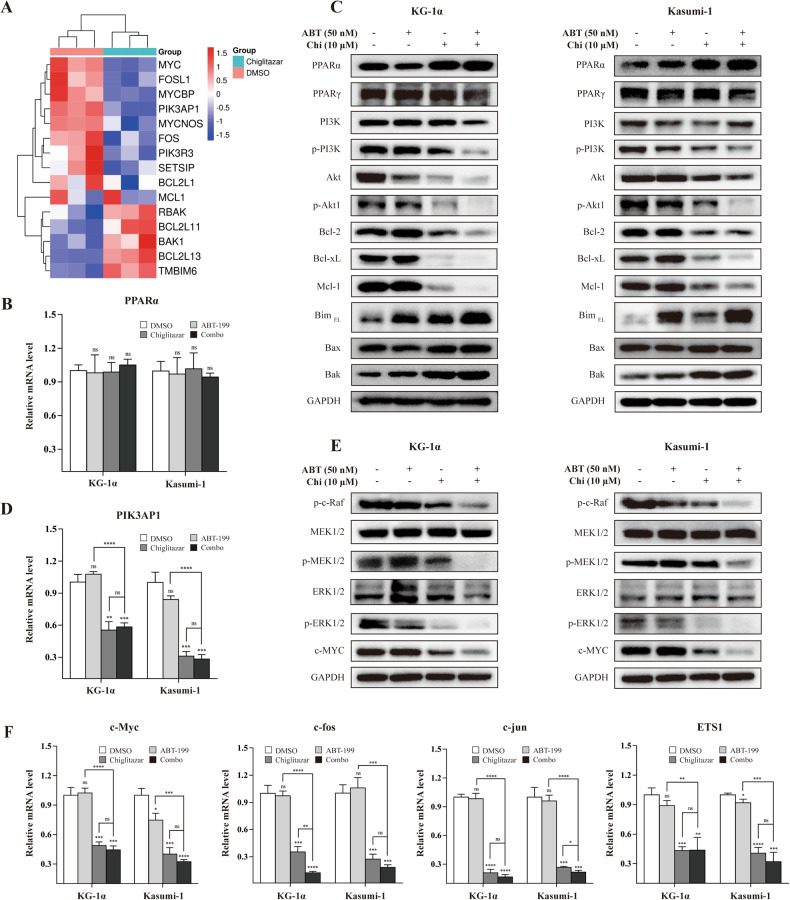
Molecular mechanisms of venetoclax combined with chiglitazar to synergistically target LSC-like cells. **A** Heat map of differentially expressed genes in KG-1α cells in response to chiglitazar for 24 h. **B** The mRNA levels of *PPARα* in KG-1α and Kasumi-1 cells treated with venetoclax and/or chiglitazar for 24 h. **C** Western blot analysis of PI3K/Akt signaling pathway and its downstream apoptotic proteins in KG-1α and Kasumi-1 cells treated with venetoclax and/or chiglitazar for 24 h. **D** The mRNA levels of *PIK3AP1* in KG-1α and Kasumi-1 cells treated with venetoclax and/or chiglitazar for 24 h. **E** Western blot analysis of the Raf-MEK-ERK pathway and c-MYC protein in KG-1α and Kasumi-1 cells treated with venetoclax and/or chiglitazar for 24 h. **F** Venetoclax combined with chiglitazar synergistically inhibited the mRNA expression of proliferation-related target genes (*c-Myc*, *c-fos*, *c-jun*, and *ETS1*) downstream of p-ERK1/2. *P < 0.05, **P < 0.01, ***P < 0.001 and ****P < 0.0001.

Next, to explore potential molecular mechanisms by which combination of venetoclax with chiglitazar in LSC-like cells, RNA sequencing analysis was performed, which showed that exposure to chiglitazar resulted in down-regulation of *PIK3AP1*, *BCL2L1* (Bcl-xL), and *MCL1*, as well as up-regulation of *BCL2L11* (Bim) and *BAK* in KG-1α cells (Fig. 3A).

*PIK3AP1* is highly expressed in most cancers, which activates the PI3K/Akt pathway [24–28]. Chiglitazar-mediated activation of the transcription factor PPARα and PPARγ is involved in the regulation of the expression of various genes [15–17]. First, we examined the expression of PPARα and PPARγ in KG-1α and Kasumi-1 cells treated with indicated agents. Although chiglitazar did not up-regulate the mRNA levels of *PPARα* (Fig. 3B), it markedly increased the protein level of PPARα, but not PPARγ (Fig. 3C). Furthermore, chiglitazar-mediated PPARα activation was associated with reduced the mRNA level of *PIK3AP1* (Fig. 3C, D).

As a specific inhibitor of Bcl-2, venetoclax is very potent in inducing apoptosis in leukemia cell lines depending on Bcl-2 [8]. We explored the potential molecular mechanisms by which venetoclax combined with chiglitazar synergistically induces apoptosis. As shown in Fig. 3C, chiglitazar-mediated activation PPARα reduced the PI3K/Akt phosphorylation and down-regulated the protein level of Akt. Moreover, exposure to venetoclax alone led to a clear increase in Bim, but not Bax and Bak. Treatment with chiglitazar alone resulted in marked down-regulation of anti-apoptotic proteins (Bcl-2, Mcl-1, and Bcl-xL) but up-regulation of pro-apoptotic proteins (Bim, Bax, and Bak). Notably, co-treatment with venetoclax and chiglitazar further down-regulation of these anti-apoptotic proteins (Fig. 3C).

These results demonstrate that chiglitazar activates PPARα, down-regulates the PI3K/Akt signaling pathway, decreases multiple anti-apoptotic proteins and increases pro-apoptotic proteins, which synergize with the Bcl-2 inhibitor venetoclax to induce apoptosis in LSC-like cells.

### Chiglitazar inhibits the proliferation-related pathways and transcription factors

Phosphorylated ERK1/2 is known to translocate from the cytoplasm to the nucleus [29], where it mediates the transcriptional activation of various proliferation-related factors such as *c-Myc*, *c-fos*, *c-jun*, and *ETS1* [30]. To further explore the potential mechanisms for inhibition of cell proliferation by chiglitazar, we examined the Raf-MEK-ERK signal pathway and found that chiglitazar alone or its combination with venetoclax clearly diminished ERK phosphorylation as well as down-regulated c-MYC protein levels (Fig. 3E). Furthermore, we found the mRNA levels of *c-Myc*, *c-fos*, *c-jun*, and *ETS1* by RT-PCR were also down-regulated (Fig. 3F). These results support that chiglitazar exerts anti-leukemia activity at least in part through inhibition of proliferation-related pathways.

### Chiglitazar-mediated PPARα activation down-regulates *PIK3AP1* in LSC-like cells

**Fig. 4:**
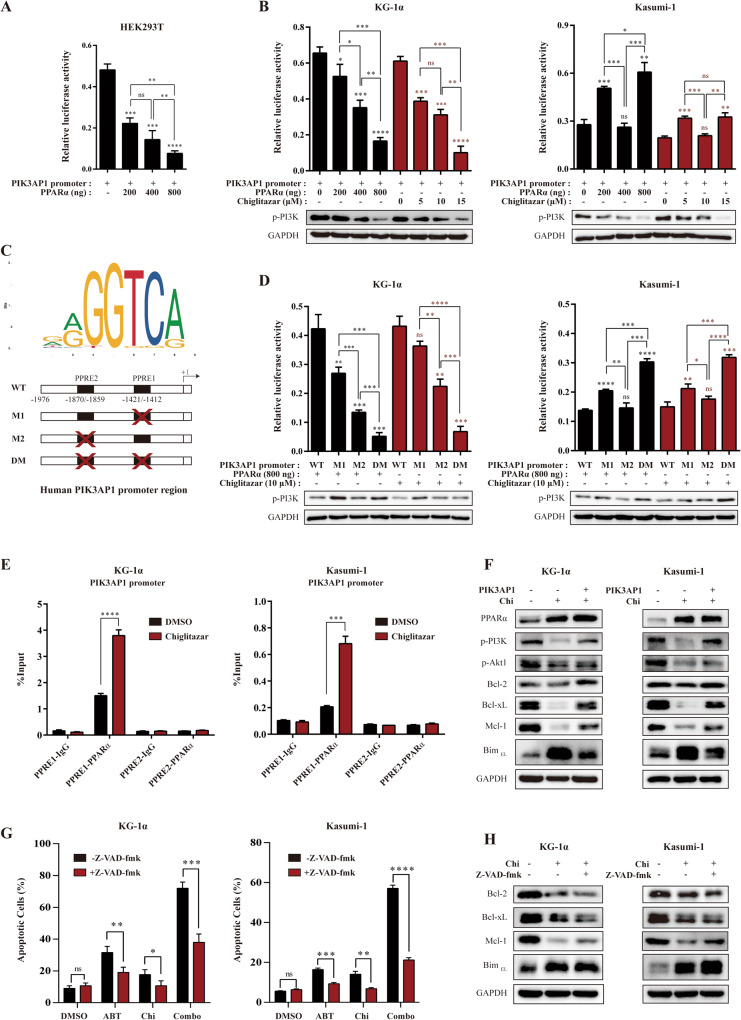
Chiglitazar-mediated PPARα activation inhibited *PIK3AP1* promoter activity in LSC-like cells. **A** The human *PIK3AP1* promoter was cloned into pGL3 and co-transfected with PPARα into HEK293T cells for dual luciferase assays. **B** KG-1α and Kasumi-1 cells were transfected with the indicated reporters with or without increasing amounts of PPARα or chiglitazar for dual luciferase assays and western blotting. **C** The binding sites for PPARα on the *PIK3AP1* promoter region was predicted using the JASPAR database. The human *PIK3AP1* promoter with deletion mutations at PPRE1 site (M1), PPRE2 site (M2), and PPRE1 and PPRE2 sites (DM) were constructed. **D** KG-1α and Kasumi-1 cells were transfected with these reporters with or without increasing amounts of PPARα or 10 μM chiglitazar for dual luciferase assays and western blotting. **E** ChIP assay analyzed the recruitment of PPARα to PPRE sites of the *PIK3AP1* gene promoter in KG-1α and Kasumi-1 cells, the chiglitazar at the concentration of 15 μM. **F** Overexpression of *PIK3AP1* in chiglitazar-treated KG-1α and Kasumi-1 cells and the experssion of PI3K/Akt phosphorylation and its downstream apoptotic proteins were analyzed by western blotting. **G** KG-1α and Kasumi-1 cells were treated with 20 μM Z-VAD-fmk for 3 h, followed by treatment with venetoclax (160 nM) or chiglitazar (16 μM) or the combination for 24 h, and then the percentages of apoptotic cells were determined by flow cytometry. **H** Western blot analysis of the BCL2 like proteins and Bim in chiglitazar-treated KG-1α and Kasumi-1 cells with/without Z-VAD-fmk. *P < 0.05, **P < 0.01, ***P < 0.001 and ****P < 0.0001.

PPARα acts as a transcription factor that recognizes and binds to RXR elements in the promoter region of target genes and regulates the expression of downstream genes [14]. We hypothesized that chiglitazar activates the transcription factor PPARα that interacts with the promoter region of *PIK3AP1* and down-regulates its expression. To test this hypothesis, we cloned the human *PIK3AP1* promoter and co-transfected HEK293T cells (Fig. 4A), KG-1α and Kasumi-1 cells (Fig. 4B) with the pGL3-PIK3AP1 promoter, pML-SV40-hRluc and various amounts of pCMV-PPARα/chiglitazar for the luciferase reporter activity assay. Transient PPARα expression significantly decreased the activity of the *PIK3AP1* promoter in a dose-dependent manner, thus inhibiting transcription and translation of this gene. Similarly, chiglitazar inhibited the transcriptional activity of the *PIK3AP1* promoter in a dose-dependent manner. To further demonstrate the functionality of these two PPRE sites, PPRE1 deletion, PPRE2 deletion and double deletions of the human *PIK3AP1* gene promoter were linked to the luciferase reporter gene (Fig. 4C) and analyzed in co-transfected KG-1α and Kasumi-1 cells (Fig. 4D). Further, ChIP assay revealed that chiglitazar increased the recruitment of PPARα to the *PIK3AP1* promoter in KG-1α and Kasumi-1 cells (Fig. 4E). Taken together, these results suggest that PPARα could be recruited to the PPRE1 site of *PIK3AP1* promoter and thus reduce the transcriptional activity of *PIK3AP1* in LSC-like cells.

To further confirm the contribution of *PIK3AP1*/PI3K signaling inhibition by chiglitazar-induced, KG-1α and Kasumi-1 cells were transfected with the pCMV-SPORT6-PIK3AP1 or empty vector and then exposed to 10 μM chiglitazar or vehicle for 24 h. The results showed that *PIK3AP1* expression rescued the expression and activation of p-PI3K and restored its downstream anti-apoptotic proteins (e.g., Bcl-2, Bcl-xL, and Mcl-1) while preventing up-regulation of Bim in chiglitazar-treated KG-1α and Kasumi-1 cells (Fig. 4F). Last, flow cytometric analysis showed the administration of the pan-caspase inhibitor Z-VAD-fmk (20 μM) significantly prevented apoptosis induced by either venetoclax and chiglitazar alone or in combination in KG-1α and Kasumi-1 cells (Fig. 4G and Supplemental Fig. S3). However, it failed to restore the expression of the anti-apoptotic proteins and prevent up-regulation of Bim in response to chiglitazar (Fig. 4H).

Collectively, our data suggest that chiglitazar-mediated PPARα activation inhibits transcriptional activity of the *PIK3AP1* gene promoter, which further induces apoptosis and inhibits cell proliferation by down-regulating PI3K/Akt signaling pathway, and cooperate with selective Bcl-2 inhibition to synergistically eliminate LSC-like cells.

### Venetoclax combined with chiglitazar synergistically inhibits the growth of CD34^+^ primary AML cells while sparing normal cells

**Fig. 5:**
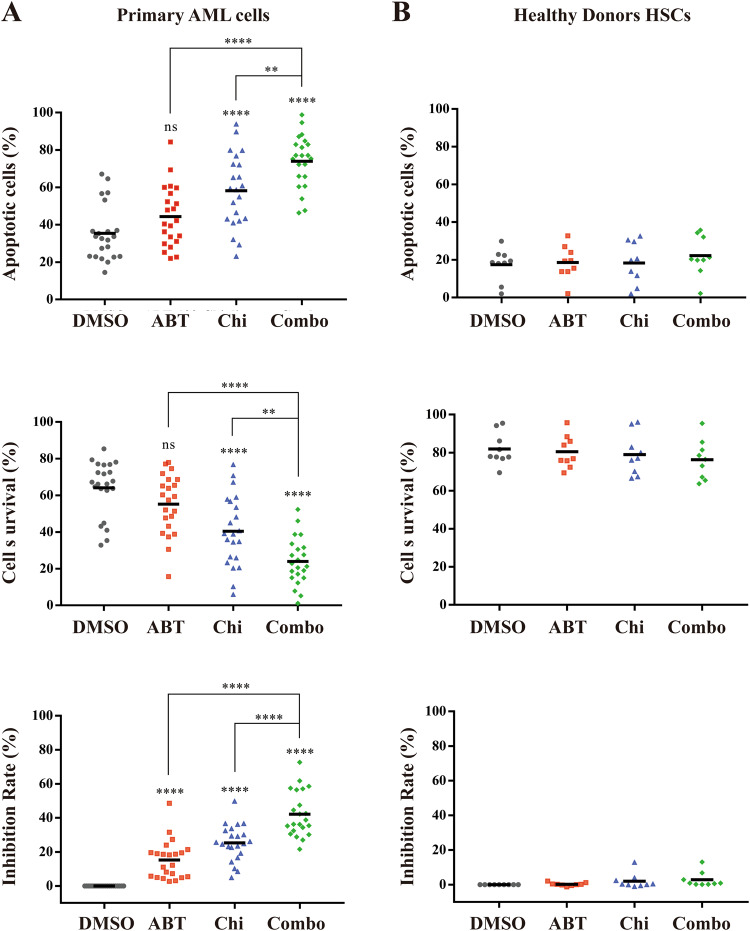
Venetoclax combined with chiglitazar preferentially targets CD34^+^ AML cells in vivo. **A** Primary AML cells were exposed to venetoclax (50 nM) or chiglitazar (10 μM) alone or in combination for 24 h, then apoptosis and survive were measured using flow cytometric analysis and CCK-8 assays (*n* = 22). **B** Healthy donors HSC cells (*n* = 9). *P < 0.05, **P < 0.01, ***P < 0.001 and ****P < 0.0001.

Next, we evaluated the anti-leukemic activity of venetoclax/chiglitazar in primary AML cells and hematopoietic stem cells (HSCs). Treatment with venetoclax or chiglitazar alone induced apoptosis (Fig. 5A; *P* = 0.0662 and *P* < 0.0001, venetoclax and chiglitazar versus DMSO, respectively). Importantly and consistent with the anti-leukemic effects observed in LSC-like cell lines, 50 nM venetoclax combined with 10 μM chiglitazar resulted significantly more robust apoptosis induction than monotherapy in CD34^+^ AML cells (Fig. 5A; *P* < 0.0001 and *P* = 0.0037, combination versus venetoclax or chiglitazar, respectively; *n* = 22). In contrast, combination treatment with the same dose of venetoclax and chiglitazar had minimal cytotoxicity in normal bone marrow CD34^+^ cells (Fig. 5B; *n* = 9). Flow cytometric data for CD34/Annexin V/PI staining of CD34^+^ primary AML cells after treatment are shown in Supplemental Fig. S4. These results show that venetoclax combined with chiglitazar has therapeutic potential to target CD34^+^ AML cells with limited toxicity toward HSCs.

### Venetoclax combined with chiglitazar exerts more effective anti-leukemia activity than monotherapy in an AML PDX model

**Fig. 6:**
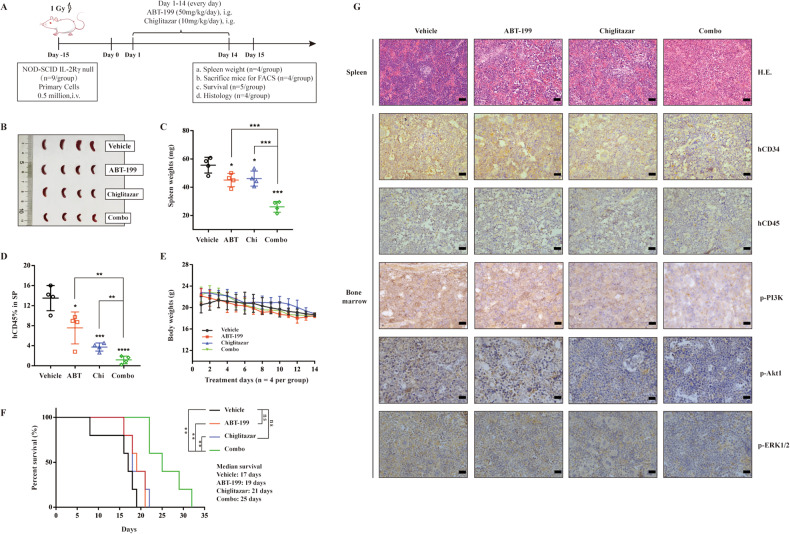
Venetoclax combined with chiglitazar suppresses tumor progression in PDX models of AML in vivo. **A** Schematic outline of the animal study. **B**, **C** Images and weights of spleens from the sacrificed mice (*n* = 4). **D** Detection of hCD45^+^ and mCD45^-^ cells in spleen was performed using flow cytometry (*n* = 4). **E** Body weight was monitored daily during treatment (*n* = 4). **F** The Kaplan-Meier survival curve was used to analyze animal survival (*n* = 5). **G** Representative data for H&E staining of the spleen and immunohistochemical staining for hCD34, hCD45, p-PI3K, p-Akt1 and p-ERK1/2 in the BM (*n* = 4). HE 200×, scale bar: 50 μm; IHC 400×, scale bar: 25 μm. *P < 0.05, **P < 0.01, ***P < 0.001 and ****P < 0.0001.

Finally, the efficacy of venetoclax/chiglitazar was tested by PDX model (Fig. 6A). ½ of the animals were analyzed on day 15 and ½ were kept for survival analysis. Based on the spleen weight and size in the different treatment groups (*n* = 4/group), venetoclax combined with chiglitazar likely improve splenomegaly associated with AML (Fig. 6B, C; all *P* < 0.05, venetoclax or chiglitazar alone versus vehicle; all *P* < 0.001, vehicle, venetoclax, or chiglitazar versus combination). As shown in Fig. 6D, hCD45^+^ cells, as determined by flow cytometry in the spleen of mice in the chiglitazar treatment group were significantly reduced compared with those in the vehicle group (*P* = 0.0003), and the proportion of hCD45^+^ cells in the spleen of mice in the venetoclax combined with chiglitazar treatment group was significantly less than that in the single treatment group (*P* = 0.008, combination versus venetoclax; *P* = 0.0037, combination versus chiglitazar) and the vehicle group (*P* < 0.0001). Supplemental Fig. S5 showed the flow cytometry data of histograms. These results showed that venetoclax combined with chiglitazar significantly reduced the tumor burden in spleen. The mouse weights slightly reduced during disease progression or with treatments (Fig. 6E), but mice behaved normal, with no obvious hair loss. Mice in the treatment groups looked more active, suggesting that the mice tolerated well to the therapy.

The remaining 5 mice in each group were followed for survival. The survival time of mice treated with venetoclax and chiglitazar monotherapy was not significant extended compared with vehicle (*P* = 0.10, venetoclax versus vehicle; *P* = 0.14, chiglitazar versus vehicle), but the combinatorial treatment significantly extended the mouse survival compared with that in the single agent treatment and vehicle group (Fig. 6F; all *P* < 0.01). The median survival time of the combined treatment group was 25 days, while the venetoclax and chiglitazar monotherapy groups and the vehicle group were 19 days, 21 days, and only 17 days, respectively.

Additionally, the histopathology analysis at 2 weeks of treatment showed that compared to that in vehicle controls, the spleen infiltration of leukemic cells of PDX mice in the venetoclax/chiglitazar treatment group was significantly reduced showing a well-preserved normal tissue structure (Fig. 6G). Finally, immunohistochemical (IHC) staining showed that the proportion of hCD34^+^ and hCD45^+^ cells in the BM of PDX mice in the combination treatment group was significantly lower than that in vehicle and single agent treatment group. The expression of p-PI3K, p-Akt1 and p-ERK1/2 was markedly down-regulated in the BM of PDX mice treated with venetoclax and chiglitazar (Fig. 6G). Together, these findings argue strongly that the regimen combining venetoclax and chiglitazar might be highly active against LSCs in vivo.

## Discussion

Although effective, most AML patients treated with the venetoclax/HMA combination therapy will develop drug resistance and clinical relapse due to various compensatory mechanisms of complex signaling networks [31–33]. High Mcl-1 and Bcl-xL levels are among the factors associating with low responses and resistance to venetoclax. LSCs are mostly in the quiescent state and have the characteristics of resistance to multiple drugs and apoptosis induction, leading to disease progression and relapse of AML patients. We aimed to develop a combination therapy and synergistic treatment scheme to enhance the antitumor activity of venetoclax and the possibility of clinical translation. Our study has demonstrated that venetoclax combined with low-cost and low-toxic chiglitazar synergistically targets AML ex vivo and in vitro.

The PI3K/Akt signaling pathway controls various cellular physiological processes, including cell differentiation, proliferation, metabolism, autophagy, angiogenesis, exocytosis and motility [34]. High PI3K/Akt signaling predicts poor prognosis in both solid tumors and hematological malignancies [35]. Previous studies have shown that *PIK3AP1*, a therapeutic target, inhibits the occurrence and development of gastric cancer [24], thyroid cancer [25], cervical cancer [26, 27], and others by regulating the PI3K/Akt signaling pathway indicating that *PIK3AP1* plays a key role in PI3K/Akt signaling. Using the JASPAR and FIMO databases, we predicted the existence of two sites (PPRE1 and PPRE2) that specifically bind to PPARα on the *PIK3AP1* promoter and experimentally confirmed the negative regulatory effect of PPARα on the *PIK3AP1* gene. However, although exposure to chiglitazar, a dual agonist of PPARα and PPARγ [17], did not alter the protein level of PPARγ, it could not be excluded that PPARγ may also play a role in anti-leukemic effect of chiglitazar alone or in combination with venetoclax.

Mitochondrial apoptosis is highly regulated by a family of pro- and anti-apoptotic Bcl-2 protein family members [36]. On the one hand, treatment with low-dose (sublethal) chiglitazar inhibited the PI3K/Akt signaling pathway by activating PPARα, which co-operated with venetoclax to regulate the expression of BCL2 family proteins (including down-regulation of anti-apoptotic Bcl-2, Bcl-xL, and Mcl-1, as well as up-regulation of pro-apoptotic Bim) in AML cells. On the other hand, the BH3-only mimetic venetoclax acts to unleash the BH3-only protein Bim from Bcl-2 to trigger the intrinsic apoptotic cascade through Bax/Bak activation [37]. However, since venetoclax does not target Mcl-1 and Bcl-xL, it cannot block binding of Bim with them and thus impairs the anti-tumor activity of this agent [38]. Therefore, one rationale is to combine the highly specific Bcl-2 inhibitor venetoclax with other agents that affect multiple other BCL2 family proteins. We found that chiglitazar down-regulated the anti-apoptotic proteins (e.g., Bcl-2, Bcl-xL, and Mcl-1) but up-regulated the pro-apoptotic proteins (e.g., Bim, Bax, and Bak) via inhibition of the PI3K/Akt pathway, therefore providing the mechanistic basis for combining it with venetoclax. Moreover, ERK phosphorylates Bim at serine 69 and Mcl-1 at threonine 163 to modulate their protein stability [39, 40], which may contribute to up-regulation of Bim and/or down-regulation of Mcl-1 in AML cells exposed to chiglitazar. Together, simultaneous neutralization of Bcl-2 (by venetoclax) and down-regulation of Bcl-2, Bcl-xL, and Mcl-1 (by chiglitazar) lead to release of Bim from these anti-apoptotic proteins, thereby inducing the activation of Bax/Bak and apoptosis. In addition, such a mechanism of action (MOA) may overcome venetoclax resistance in AML cells [41, 42].

The cross-talk of PI3K and MAPK (mitogen-activated protein kinase) pathway has been reported previously. For example, several studies have shown that PI3K can indirectly activate the Raf/MEK/ERK signaling cascade in AML [43–45]. The latter pathway is often constitutively activated in AML, leading to a specific expression profile of genes that determines cell fate [46]. The ERK1/2 phosphorylation at Thr183 and Tyr185 activates the protein, which then enters the nucleus to regulate the transcription of growth factors [47]. Consistently, we found that chiglitazar, a PPARα agonist, down-regulated the expression of p-c-Raf, p-MEK and p-ERK, thereby inhibiting the transcription of growth factors such as *c-Myc*, *c-fos*, *c-jun*, and *ETS1* and thus suppressing cell growth and proliferation.

**Fig. 7:**
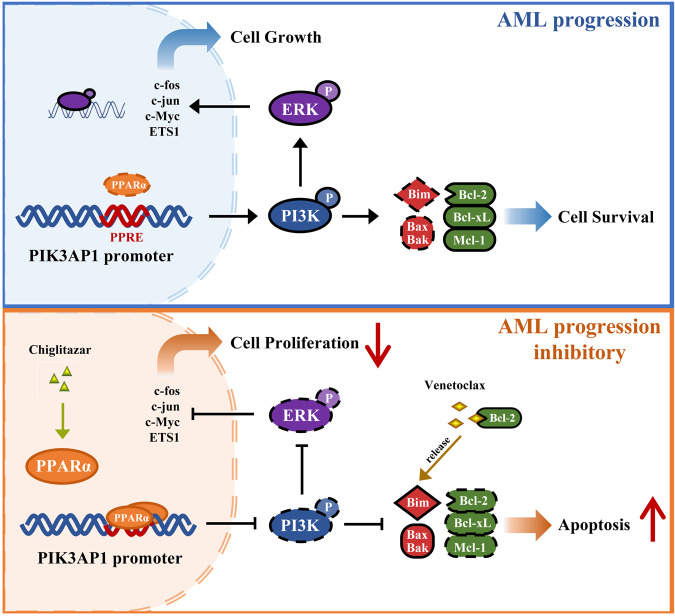
Mechanism of action of the combined treatment. A schematic model for the mechanism by which venetoclax synergistically interacts with chiglitazar to inhibit AML progression.

Furthermore, this study demonstrated that venetoclax combined with chiglitazar resulted in synergistic lethality to primary CD34^+^ AML cells while largely sparing their normal counterparts. While the exact mechanism for such selectivity remains unclear, one possible explanation is the differential expression of PPARα between HSCs and CD34^+^ AML cells [48]. The efficacy of this regimen was further validated in a PDX model. The model was generated from LSCs of a refractory relapsed AML patient and it was frequently used for the preclinical evaluation of personalized treatment strategies [49, 50]. In conclusion, we demonstrate that PPARα activation by chiglitazar synergizes with Bcl-2 inhibition by venetoclax against LSC-like cells at least in part through suppressing the transcriptional activity of the *PIK3AP1* gene promoter, down-regulating the PI3K/Akt signaling pathway, and decreasing multiple cell survival proteins (Fig. 7). This study provides strong preclinical evidence that the combination regimen of venetoclax and chiglitazar may be an alternative treatment for AML patients with refractory/relapsed diseases, especially those who do not meet the conditions of intensive chemotherapy and lack of responses to venetoclax-based therapies.

## Materials and methods

### Reagents and cells

Venetoclax and chiglitazar were all purchased from Chipscreen Bioscience Ltd. (Shenzhen, China) and dissolved in dimethyl sulfoxide (DMSO) for all in vitro assays.

KG-1α and Kasumi-1 cell lines were kindly provided by Union Hospital, Fujian Medical University (Fuzhou, China) and cultured in RPMI-1640 medium supplemented with 10% FBS, 100 μg/mL streptomycin and 100 U/mL penicillin (HyClone, Thermo Scientific, MA, USA) in an environment containing 5% CO_2_ at 37 °C. The HEK293T cells were purchased from ATCC (Teddington, UK) and cultured in DMEM medium with the same conditions.

### Cell sorting

CD34^+^/CD38^-^-enriched cells were sorted form KG-1α and Kasumi-1 cells using flow cytometry (FACS Aria IIU, BD) after staining with anti-CD34-APC and anti-CD38-PE. In addition, cells were stained with anti-CD123-PE, anti-CD96-PE, anti-CD25-PE, anti-CD44-FITC, anti-CD32-APC, and anti-CD47-Brilliant Violet 421 (Biolegend, CA, USA) for 30 min at 4 °C and then subjected to flow cytometric analysis for documenting their LSC phenotypic features.

### Patient samples

Human bone marrow (BM) samples were obtained from patients following the informed consent in accordance with the Declaration of Helsinki and protocols approved by the Ethics Review Board of First Affiliated Hospital of Xiamen University. HSCs and primary AML cells were separated from the BM of healthy donors and AML patients using Ficoll-Hypaque density gradient column (Cytiva, Uppsala, Sweden), respectively. Primary CD34^+^ AML cells were sorted using flow cytometry as described above.

### Cell proliferation and viability assays

A total of 2 × 10^5^/mL KG-1α or Kasumi-1 cells were inoculated and treated with DMSO, the indicated doses of venetoclax and chiglitazar alone or in combination for 24 h. Then trypan blue (MCE, Shanghai, China) was used to measure the cell number at various time points.

A total of 1 × 10^4^ KG-1α or Kasumi-1 cells were seeded in 96-well plates containing 100 μL medium and treated with designated drugs for 24 h. After treatment, the CCK-8 kit (MCE) was used to measure cell viability. CompuSyn software and the Chuou-Talalay medium effect analysis method were used to calculate the fraction affected (Fa) and the corresponding drug combination index (CI) of each treatment group and to draw the Fa-CI curve [51].

### Flow cytometric analysis of apoptosis

To assess apoptosis, 5 × 10^5^ KG-1α or Kasumi-1 cells were inoculated in 24-well plates containing 1 mL medium and treated with designated drugs for 24 h, followed by staining with Annexin V/PI (Biolegend) in dark for 15 min. Then, flow cytometry was used to analyze the percentage of apoptotic cells (Annexin V^+^).

### Clonogenic assay

Cell colony formation was evaluated as described previously [52]. A total of 5 × 10^5^/mL KG-1α or Kasumi-1 cells were treated with DMSO, 100 nM venetoclax and 10 μM chiglitazar alone or in combination for 24 h. Subsequently, 1000 cells from every treatment were incubated in 6-well soft agar and cultured for 14 days at 37 °C. Colonies were stained with 0.5% crystal violet stain solution (MCE) and CFUs consisting of at least 50 cells were counted.

### RNA-sequencing

A total of 1 × 10^7^ KG-1α cells were incubated with chiglitazar (15 μM) for 24 h, followed by total RNA extraction with TRIzol reagent (Invitrogen). RNA-seq experiment and high through-put sequencing and data analysis were conducted by Seqhealth Technology Co., LTD (Wuhan, China).

### RNA isolation and qPCR

A total of 1 × 10^6^ KG-1α or Kasumi-1 cells were seeded in 6 cm plates containing 4 mL medium and treated with DMSO, 50 nM venetoclax and 10 μM chiglitazar alone or in combination for 36 h. The TRIzol reagent was used to extract and purify the total RNA, 1 µg of which were reverse transcribed to cDNA using Evo M-MLV RT Premix (Accurate Biology, Hunan, China). Then, the qPCR was performed with SYBR Green Premix Pro Taq HS qPCR Kit (Accurate Biology). The 2^−ΔΔCT^ method was used for analysis and GAPDH was used as an endogenous reference. Relevant primers are listed in Supplemental Table S1.

### Western blot analysis

Western blot analysis was performed as previously described [53]. The primary antibodies were as follows: PPARα (ab227074), PPARγ (ab178866), PI3K (#4292), p-PI3K (#4228), Akt (#4685), p-Akt1 (AP0637), Bcl-2 (#15071), Bcl-xL (#2762), Mcl-1 (#39224), Bim (A19702), Bax (#2774), Bak (#12105), p-c-Raf (#9431), MEK1/2 (A18117), p-MEK1/2 (#9154), ERK1/2 (A10613), p-ERK1/2 (#3510), c-MYC (#18583), and GAPDH (#2118).

### Plasmid construction

The sequence of the *PIK3AP1* promoter was found in the UCSC database (https://genome.ucsc.edu/) and was compared and validated in NCBI. The binding site of PPARα in the *PIK3AP1* promoter region was predicted by JASPAR database (https://jaspar.genereg.net/).

Human *PIK3AP1* promoter fragments were amplified by PCR from human genomic DNA with the primers 5′-GGTACCAGTTGTCCAGATGCACTTGGTT-3′ and 5′-AAGCTTCTGGCGCTTCCTCCCTCA-3′. Deletion mutations were made to the binding sites in the promoter region using the overlap PCR method. The PCR products were digested by *Kpn* I and *Hind* III, and cloned into the reporter plasmid pGL3-Basic (Promega Corp., Madison, WI, USA) using T4 ligase to generate the pGL3-PIK3AP1 promoter-LUC. Similarly, human *PPARα* (5′-GCGTGCACGGCACAACCAGCACCAT-3′ and 5′-CGGGATCCTGCTCCCCCGTCTCCTTTG-3′) and human *PIK3AP1* (5′-AGATCTATGGCAGCCTCAGGGGT-3′ and 5′-GGTACCTCAGCGTCCTCTGGGTGG-3′) were cloned into the pCMV vector (Promega) to generate pCMV-3× FLAG-PPARα and pCMV-SPORT6-PIK3AP1, respectively.

### Transfection and luciferase reporter assay

The pGL3-PIK3AP1 promoter-LUC, PML-SV40-HRLUC and pCMV-3×FLAG-PPARα were co-transfected into HEK293T cells using Lipofectamine 2000 (Thermo Fisher Scientific, Waltham, MA) for 36 h and into KG-1α and Kasumi-1 cells by electrotransfection (Bio-Rad, California, USA), respectively. These cells were washed with pre-cooled 1×PBS and lysed with Harvest Buffer (200 μL/per well) for 10 min. After being centrifuged, the supernatant was mixed with the luciferase substrate, and the fluorescence intensity was measured within 30 s. Firefly luciferase activity values were divided by *Renilla* luciferase activity values to obtain the normalized luciferase activities (Promega).

### Chromatin immunoprecipitation (ChIP) assay

ChIP assay was performed as previously described [54]. Briefly, 2 × 10^7^ KG-1α or Kasumi-1 cells were incubated with 15 μM chiglitazar for 24 h. The ChIP assay was performed using SimpleChIP Enzymatic Chromatin IP kit (Cell Signaling Technology) and detected by quantitative PCR using the following primers targeting specific region of the *PIK3AP1* promoter, including PPRE1: 5′-AGCTGGGATGGTCTCGATCTC-3′ and 5′-CTTCTGTCTCCCTCTTCCAG-3′; PPRE2: 5′-TCCCTAACATCCGGAGCTGG-3′ and 5′-GGCCTGGCCAGTTTGTGTT-3′.

### Patient-derived xenograft (PDX) models

All animal experiments were approved by Xiamen University Animal Ethics and Management Committee. NOD-SCID IL-2Rγ null (NSG) mice (4–6 weeks old, female) were bred with specific pathogen-free (SPF) conditions. To generate PDX mouse models, these NSG mice were injected with 5 × 10^5^ humanized splenocytes from xenograft mice established by administering primary CD34^+^ AML cells via tail vein after irradiation with 1 Gy. Cells were obtained from a patient with newly-diagnosed AML (female, 38 years old, FAB M5, 1.4 × 10^9^/L WBC, with mutations of *DNMT3A*, *IDH1*, and *NPM1*), without prior treatments (including venetoclax or chiglitazar). After 14 days, these mice were randomly assorted to four groups (*n* = 9/group) and treated with vehicle, venetoclax (50 mg/kg/day, oral gavage), chiglitazar (10 mg/kg/day, oral gavage) or the combination for two consecutive weeks.

These mice were monitored and weighed daily for toxicity (*n* = 4) during treatment. After treatment, these mice were euthanized and the spleen weight was measured with an analytical balance. Then, the percentage of human CD45^+^/mouse CD45^-^ cells in spleen was measured by flow cytometry (*n* = 4). Since leukemia burden in spleen is defined as hCD45^+^ and mCD45^-^ cells, hCD45 cells were detected by human CD45 antibodies and mCD45 cells were detected with murine CD45 (Biolegend). The survival of the remaining mice was monitored (*n* = 5).

### Immunohistochemistry

Immunohistochemistry (IHC) and hematoxylin and eosin staining (H&E staining) were performed as previously described [53]. Briefly, tissue slides were incubated with the primary antibodies against human CD34 (ab110643), human CD45 (#13917), p-PI3K (#4228), p-Akt1 (AP0637) and p-ERK1/2 (AP0974).

### Statistical analysis

One-way or two-way analysis of variance (ANOVA) respectively followed by post-hoc test of the LSD or Bonferroni with GraphPad Prism 6 Software (USA) were used to assess statistical analysis. All date represent the mean ± standard deviation (SD) for at least three independent experiments. *P* < 0.05 is considered statistically significant differences between groups.

## Supplementary information

SUPPLEMENTAL MATERIAL

SUPPLEMENTAL MATERIAL

aj-checklist

## Data Availability

The datasets presented in this study are available from the corresponding authors with reasonable requests.
