# Supplementary material for: Bcl-2 inhibition combined with PPARα activation synergistically targets leukemic stem cell-like cells in acute myeloid leukemia
Source: Cell Death Dis. 2023 Aug 29;14(8):573. doi: 10.1038/s41419-023-06075-6 (PMC10465498; doi:10.1038/s41419-023-06075-6)
Supplement: Supplementary file 1 — SUPPLEMENTAL MATERIAL [file 41419_2023_6075_MOESM1_ESM.docx]

**Supplemental Information**

**Supplemental Table S1**

| Primer sequences of genes used for quantification of mRNAs by real-time PCR | |
| --- | --- |
| GAPDH | 5’-GCTCATTTGCAGGGGGGAG-3’ |
|  | 5’-GTTGGTGGTGCAGGAGGCA-3’ |
| PPARα | 5’-TCTGGCCAAGAGAATCTACGAG-3’ |
|  | 5’-CAGCCATACACAGTGTCTCCAT-3’ |
| PIK3AP1 | 5’-TCATCGTCTACAGCCCGGAT-3’ |
|  | 5’-TCAGTATCTTCTGGCTGCGG-3’ |
| c-Myc | 5’-GCTGCTTAGACGCTGGATTT-3’ |
|  | 5’-CACCGAGTCGTAGTCGAGGT-3’ |
| c-fos | 5’-TCTTCCTTCGTCTTCACC-3’ |
|  | 5’-AATCAGAACACACTATTGCC-3’ |
| c-jun | 5’-GCCTACAGATGAACTCTTTCTGGC-3’ |
|  | 5’-CCTGAAACATCGCACTATCCTTTG-3’ |
| ETS1 | 5’-TAAGTGAGGTGCTGAGAGCAG-3’ |
|  | 5’-CCCAAAAGGGGTAGCAAGGT-3’ |

**
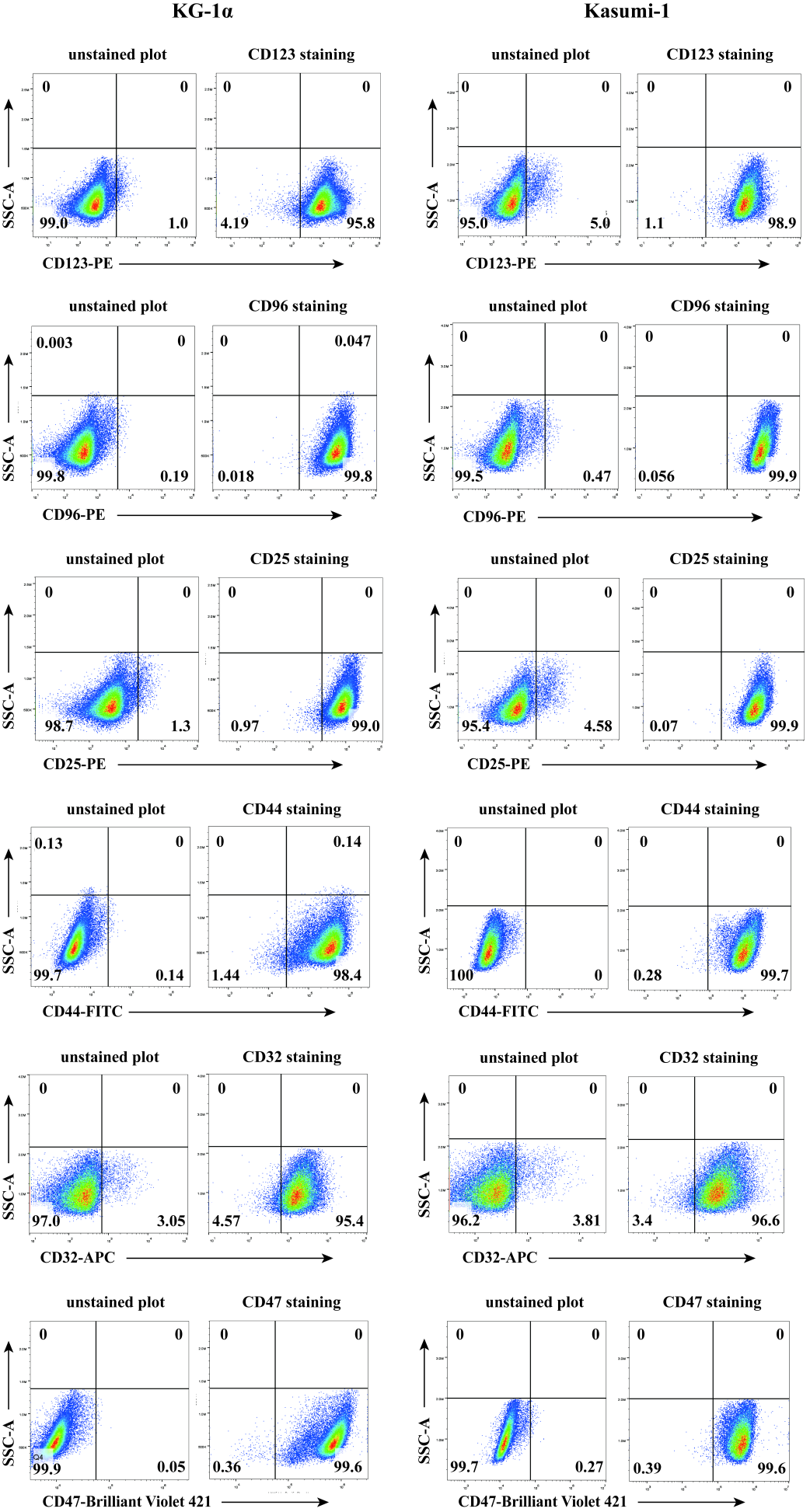
**

**Supplemental Fig. S1** Representative FACS analysis of the CD123^+^, CD96^+^, CD25^+^, CD44^+^, CD32^+^, and CD47^+^ percentage among KG-1α and Kasumi-1 cells.**
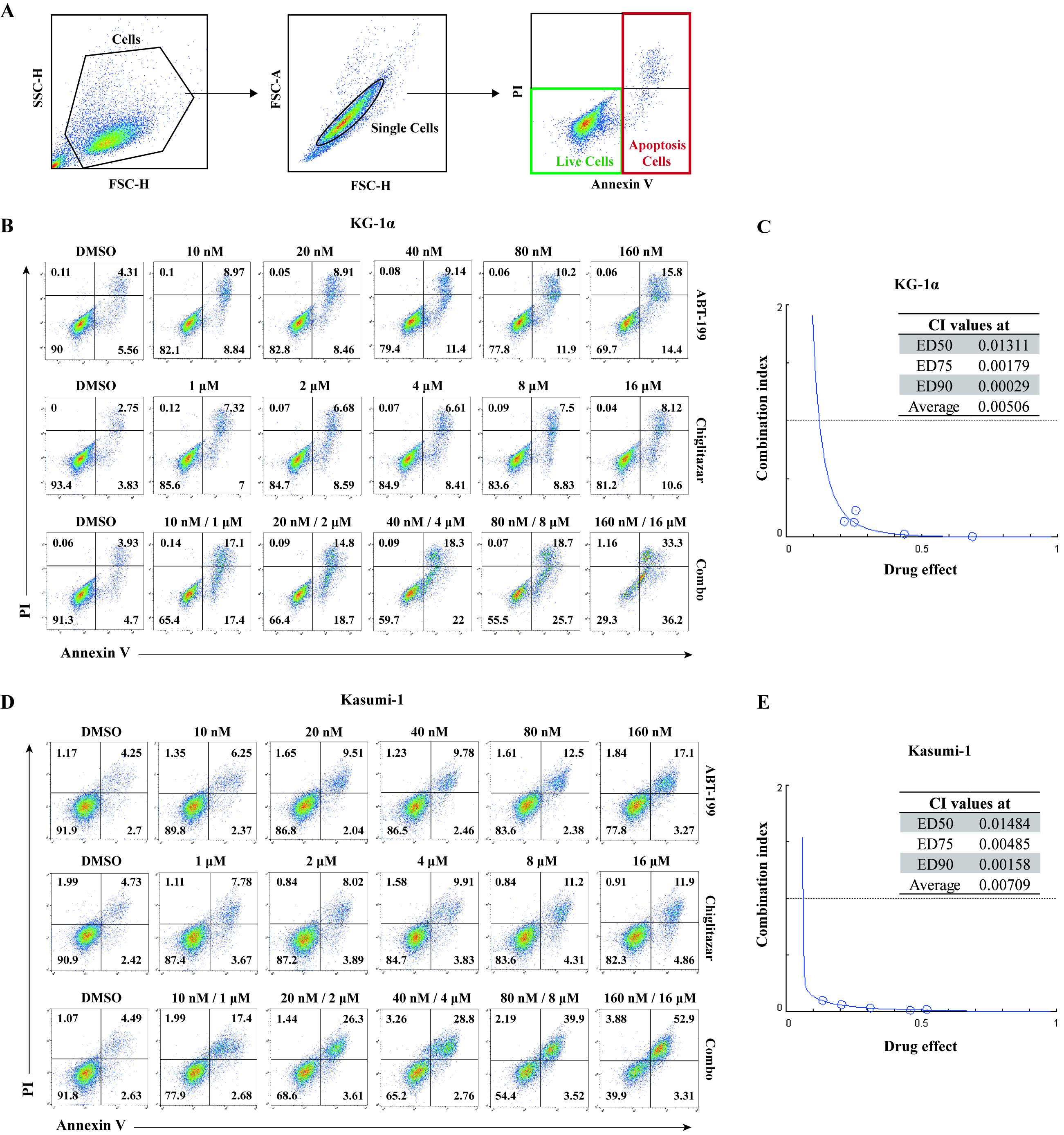
**

**Supplemental Fig. S2** **A** Flow cytometric plots showing the gating strategy to determine the percentages of apoptotic cells (Annexin V^+^) and live cells (Annexin V^−^/PI^−^). **B-E** The combination of venetoclax and chiglitazar synergistically induced apoptosis and combination index (CI) plots in KG-1α and Kasumi-1cells in vitro.


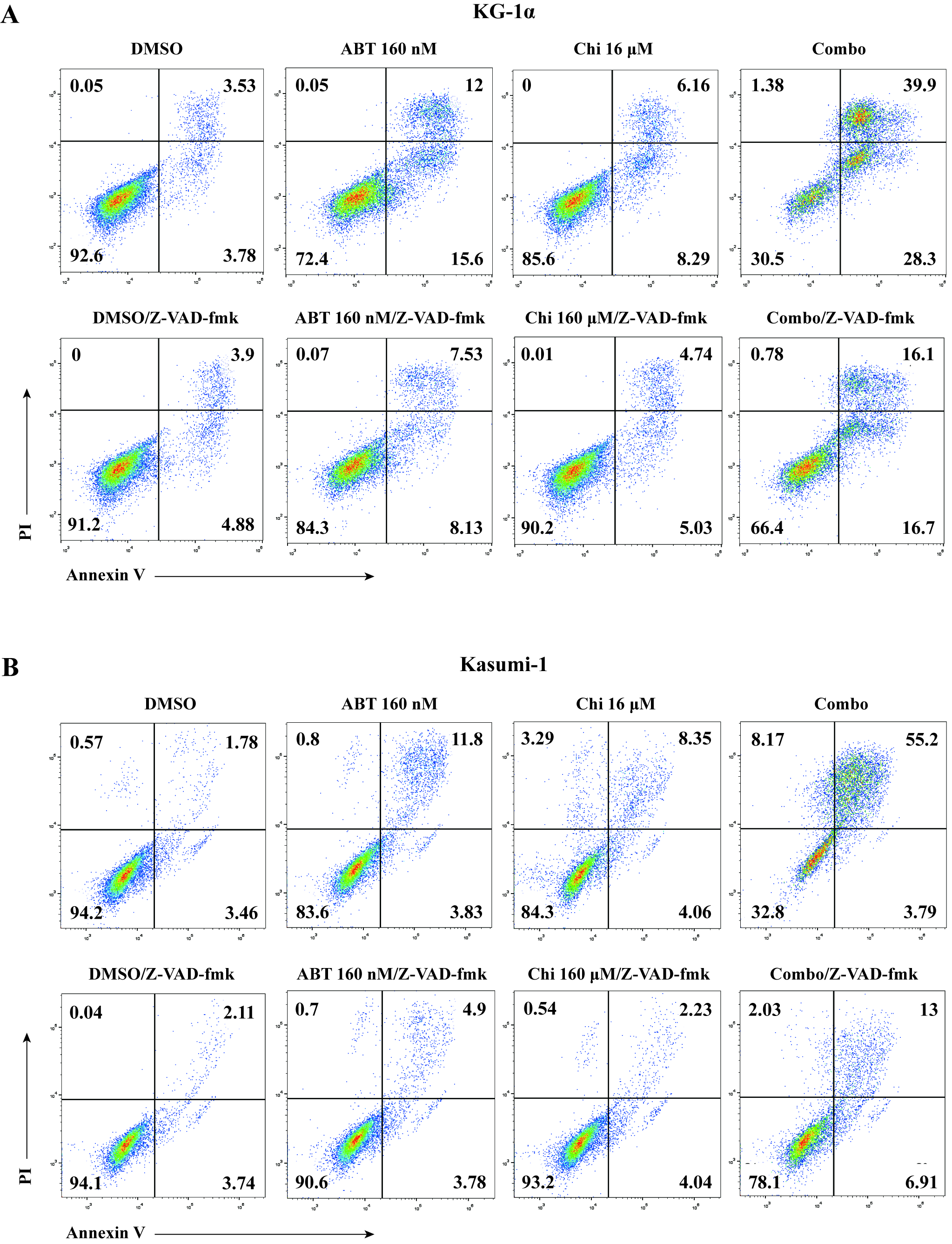


**Supplemental Fig. S3** Representative flow cytometric data for Annexin V/PI staining in drug-treated KG-1α and Kasumi-1 cells after Z-VAD-fmk (20 μM) pretreatment.


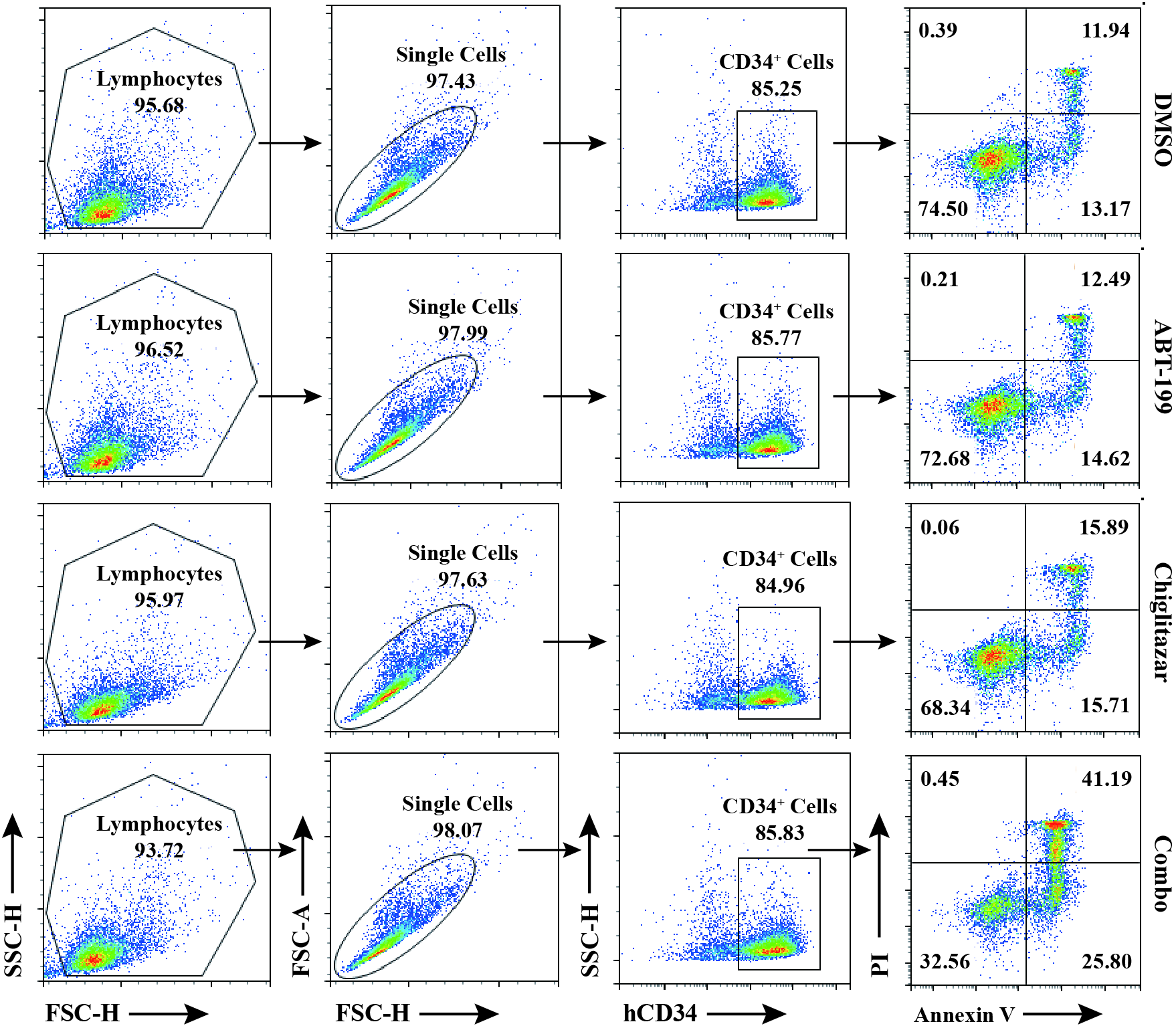


**Supplemental Fig. S4** Representative flow cytometric data for hCD34/Annexin V/PI staining in primary CD34^+^ AML cells after venetoclax/chiglitazar treatment.


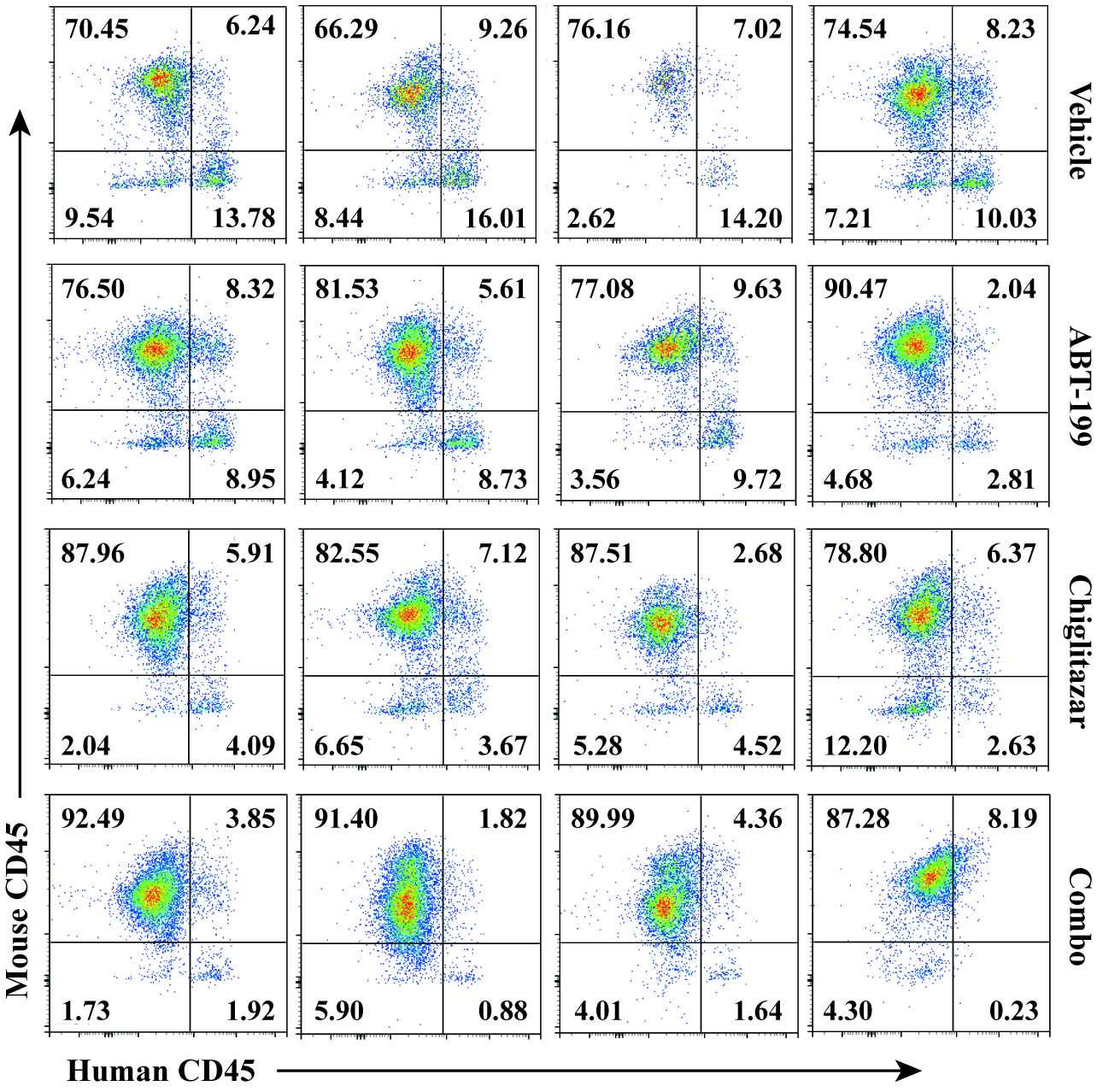


**Supplemental Fig. S5** Representative data for flow cytometric analysis of hCD45/mCD45 staining in spleen.
