# Supplementary material for: Bcl-2 inhibition combined with PPARα activation synergistically targets leukemic stem cell-like cells in acute myeloid leukemia
Source: Cell Death Dis. 2023 Aug 29;14(8):573. doi: 10.1038/s41419-023-06075-6 (PMC10465498; doi:10.1038/s41419-023-06075-6)
Supplement: Supplementary file 2 — SUPPLEMENTAL MATERIAL [file 41419_2023_6075_MOESM2_ESM.docx]

**Fig. 3C**

PPARα
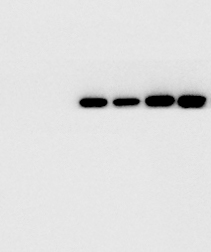
 PPARα
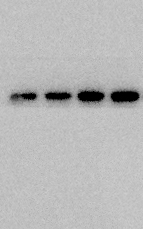


PPARγ
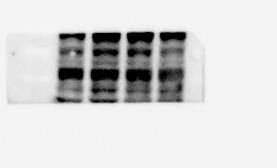
 PPARγ
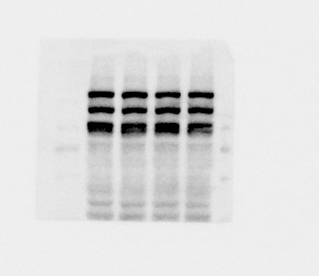


PI3K
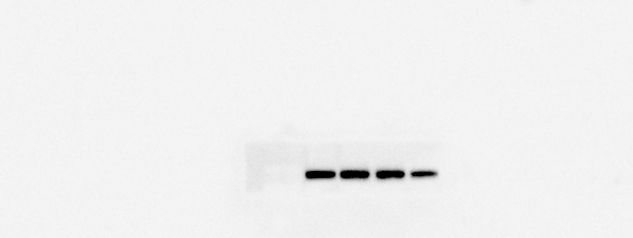
 PI3K
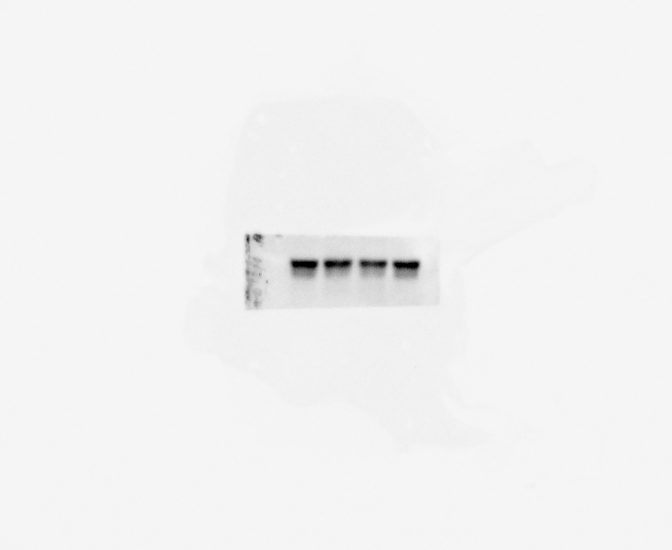


p-PI3K
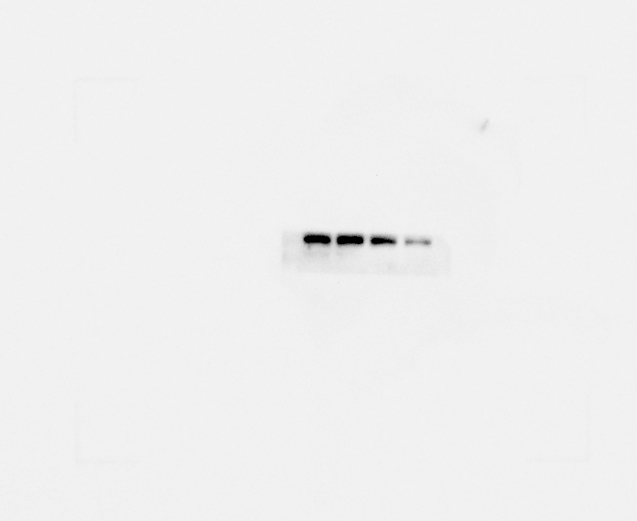
 p-PI3K
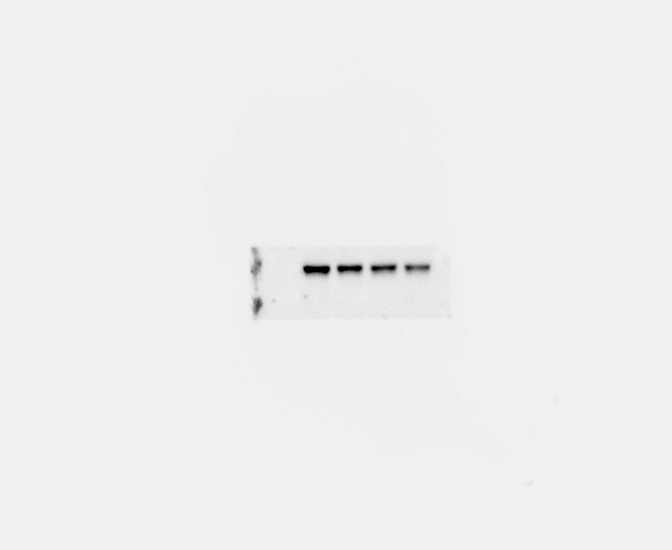


Akt
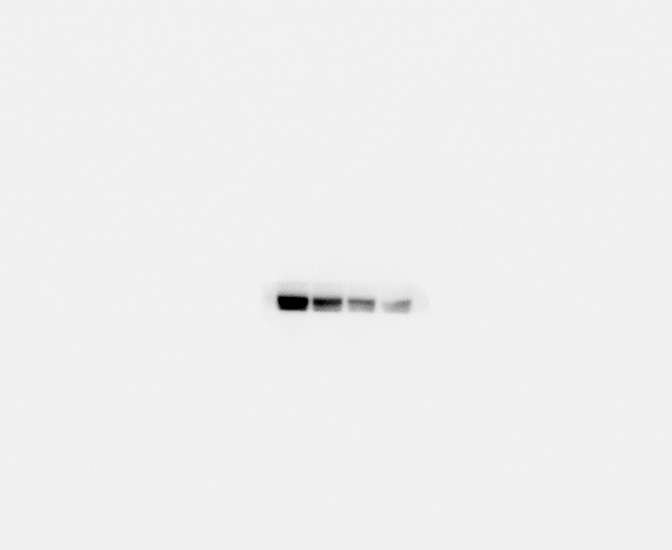
 Akt
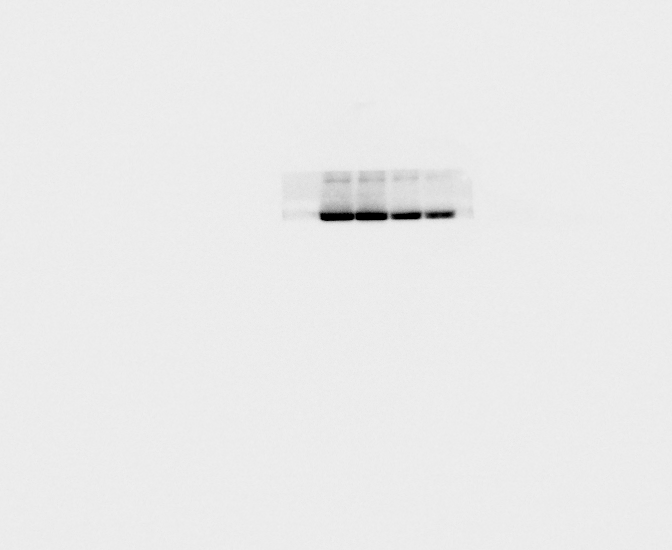


p-Akt1
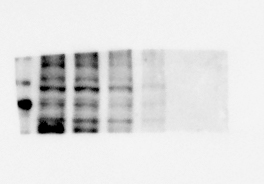
 p-Akt1
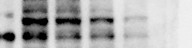


Bcl-2
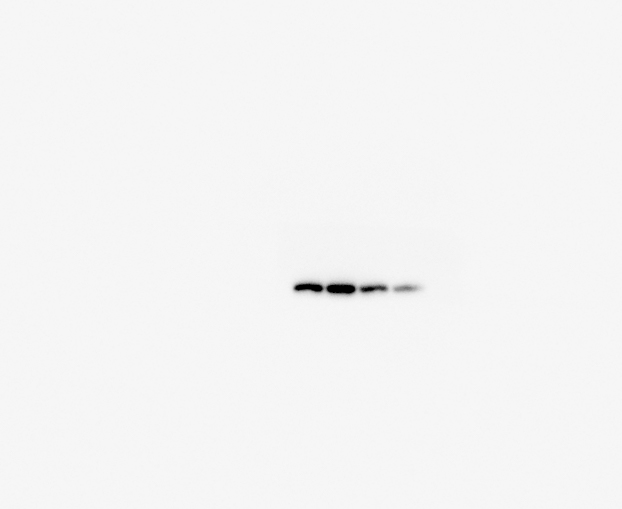
 Bcl-2
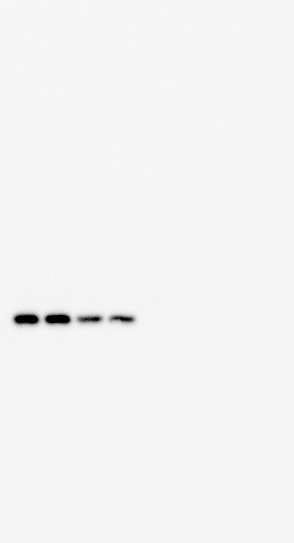


Bcl-xL
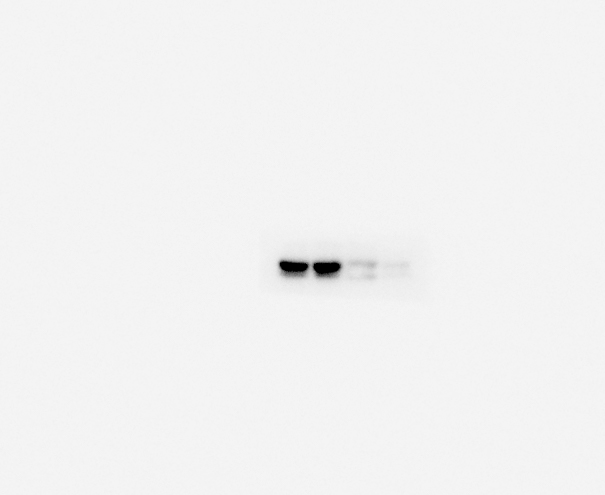
 Bcl-xL
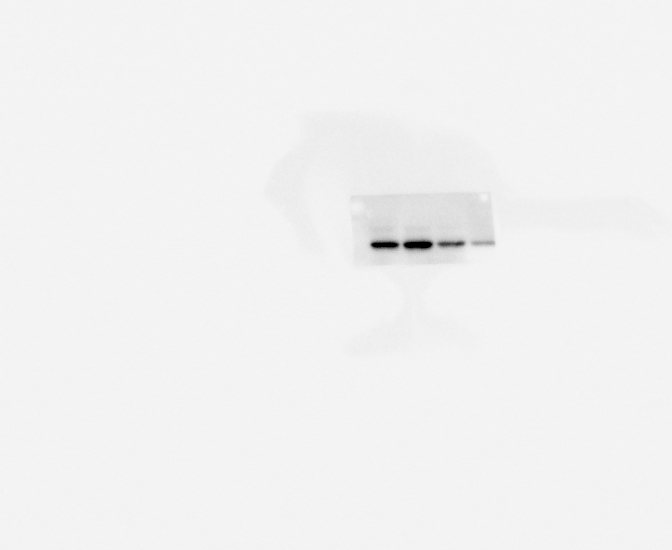


Mcl-1
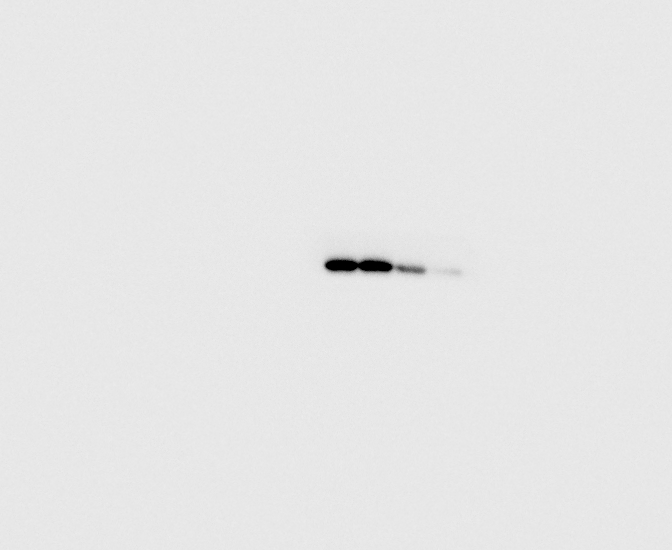
 Mcl-1
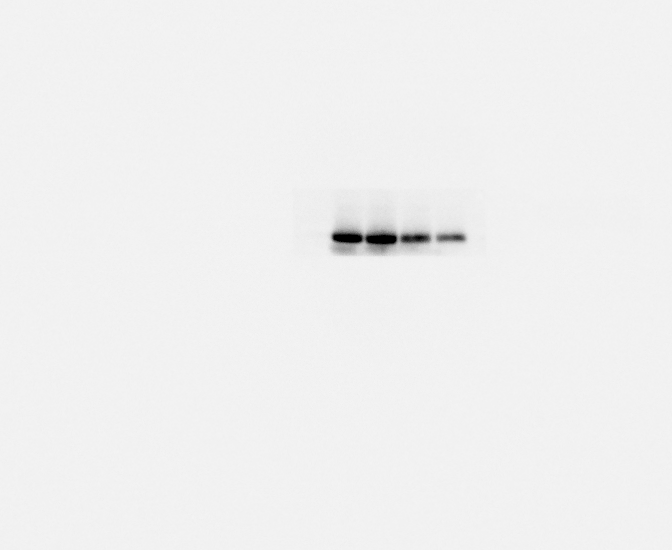


Bax
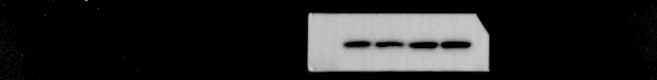
 Bax
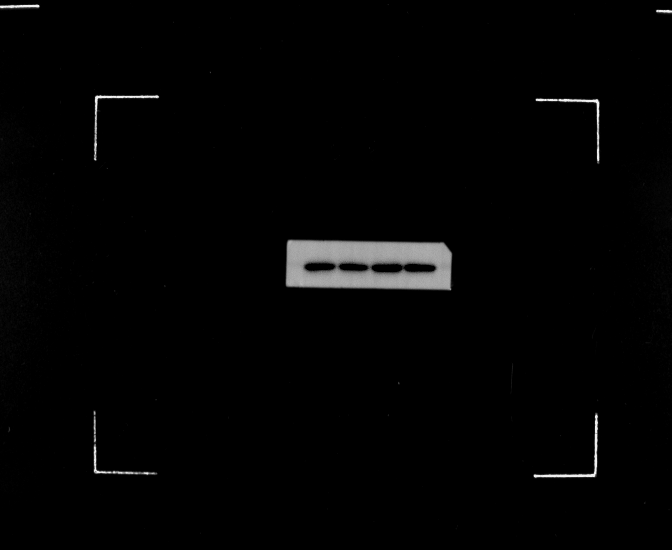


Bak
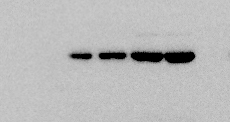
 Bak
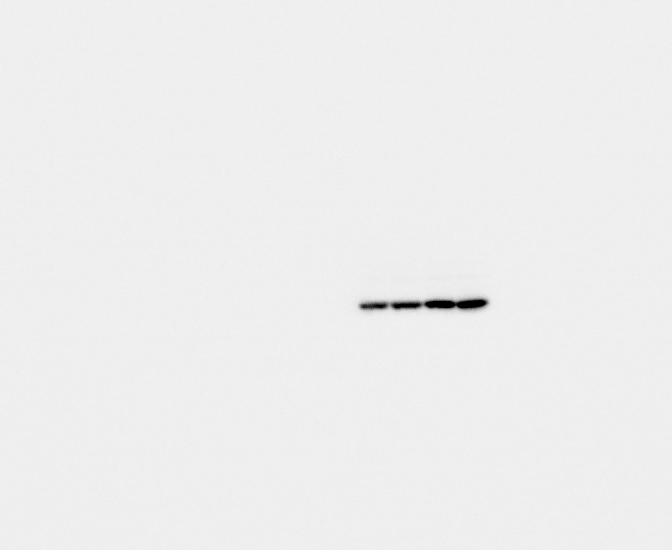


Bim
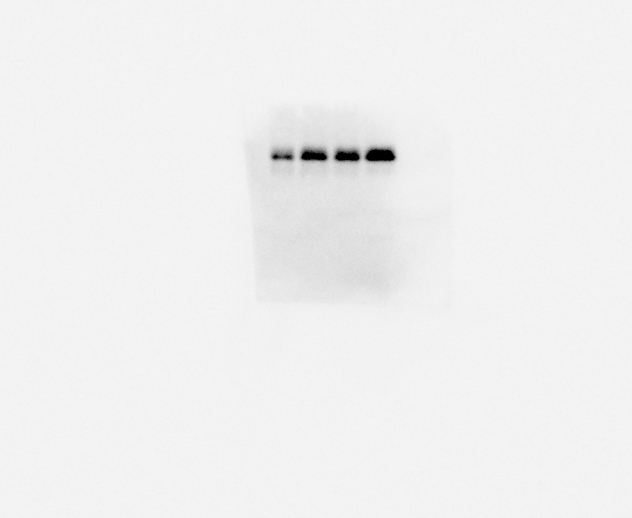
 Bim
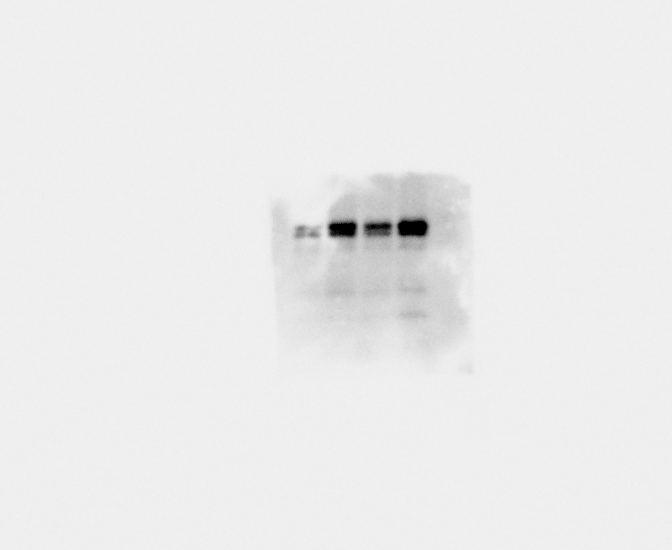


GAPD
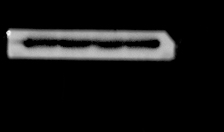
 GAPDH
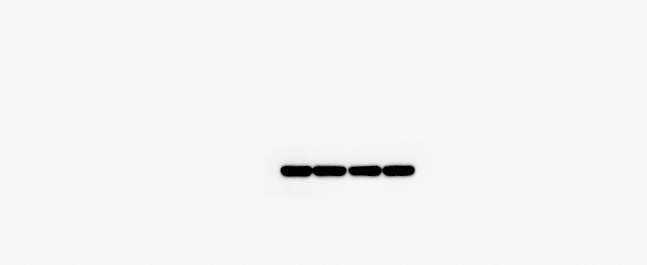


**KG-1α Kasumi-1**

**Fig. 3E**

p-c-Raf
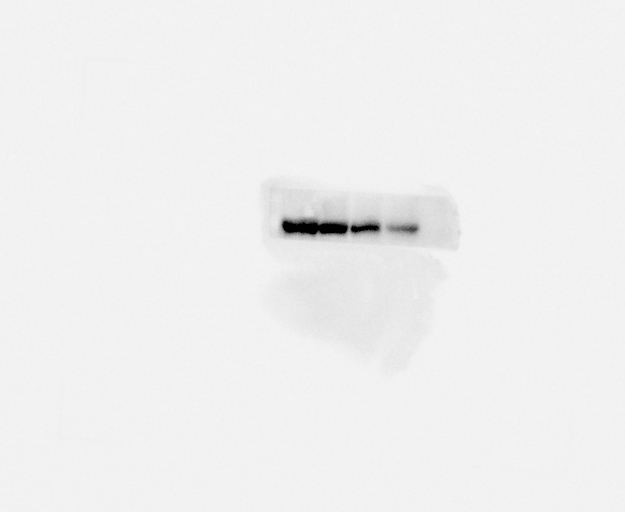
 p-c-Raf
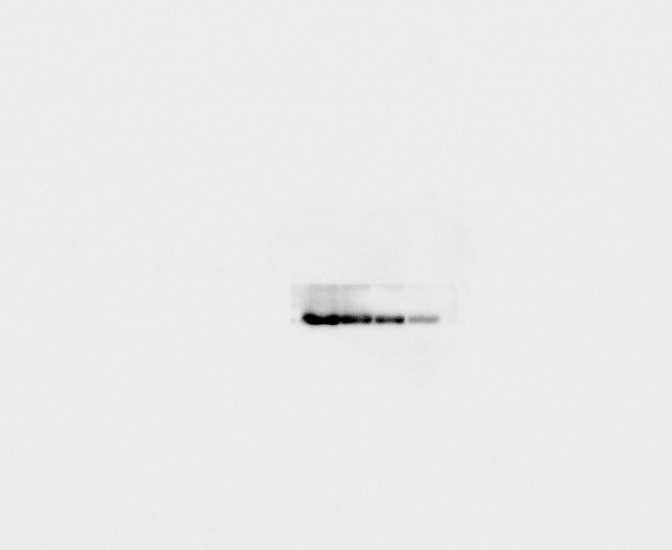


MEK1/2
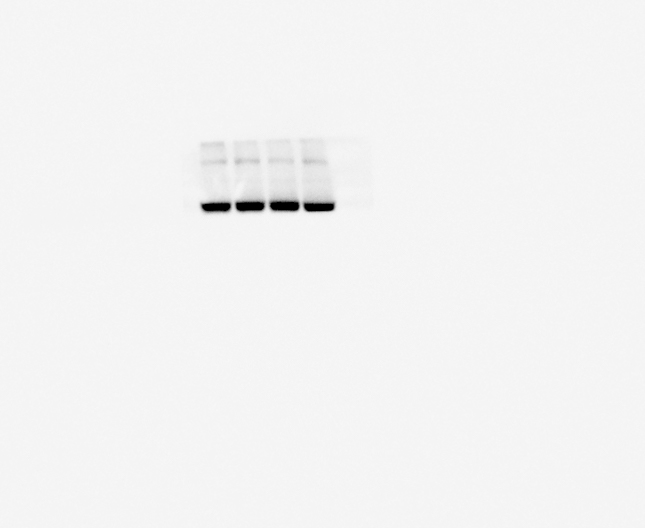
 MEK1/2
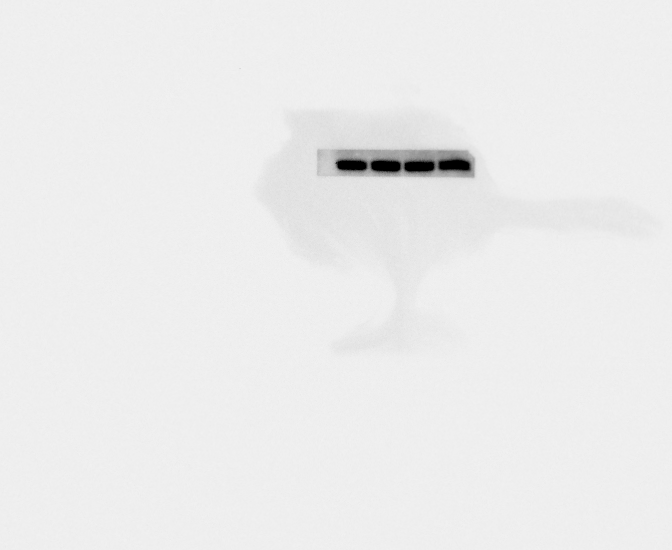


p-MEK1/2
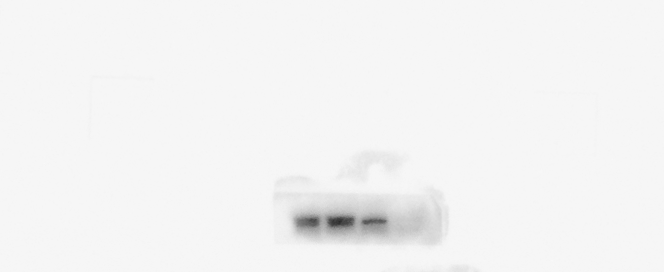
 p-MEK1/2
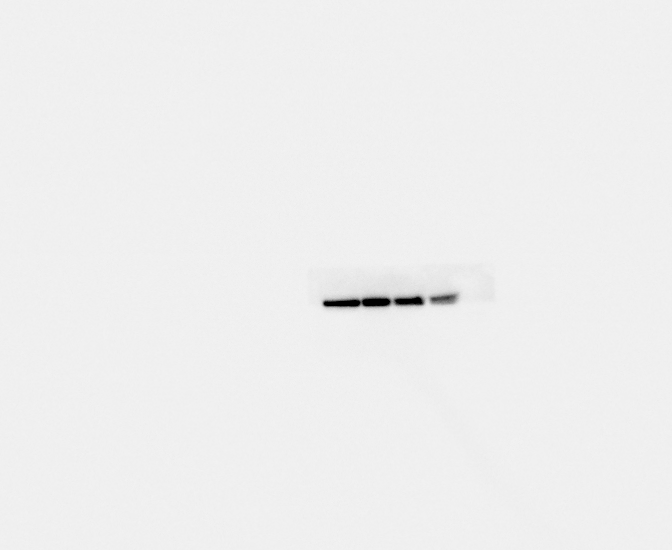


ERK1/2
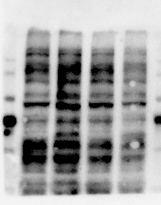
 ERK1/2
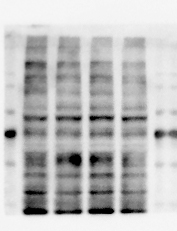


p-ERK1/2
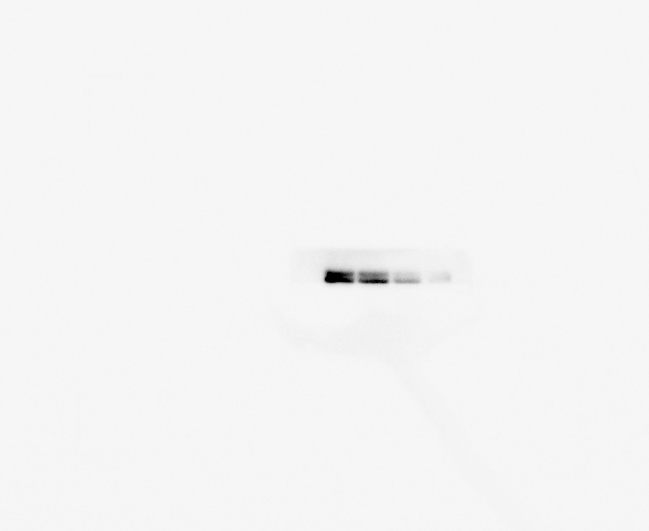
 p-ERK1/2
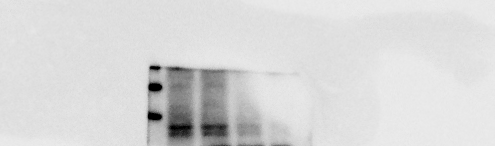


c-MYC
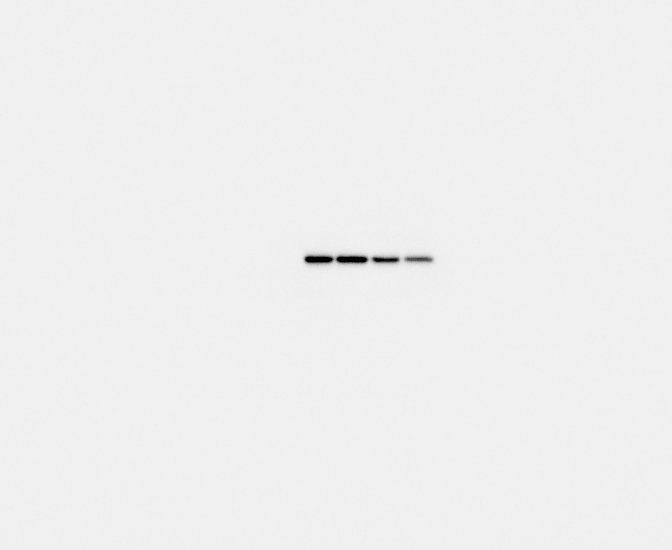
 c-MYC
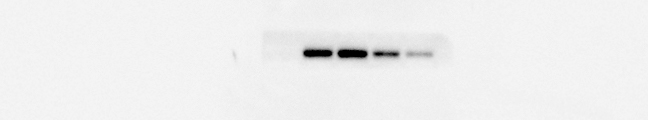


GAPDH
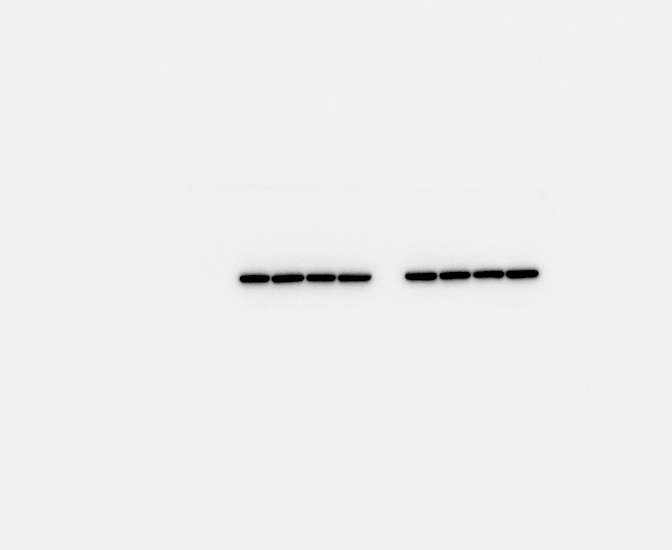
 GAPDH
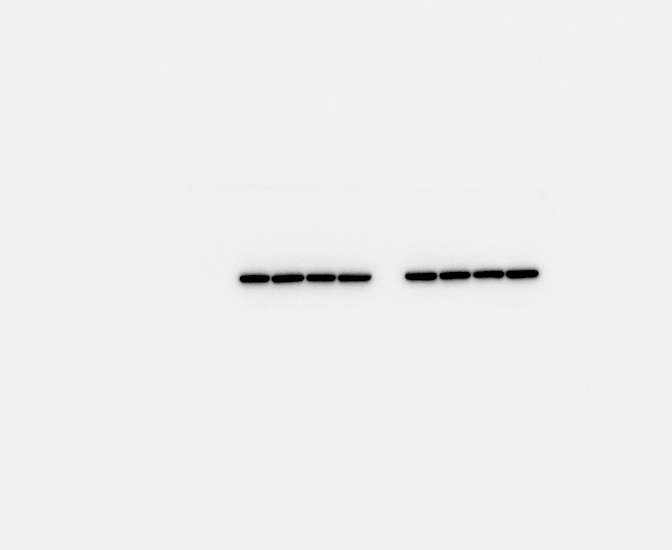


**KG-1α Kasumi-1**

**Fig. 4B**

**KG-1α**: p-PI3K **
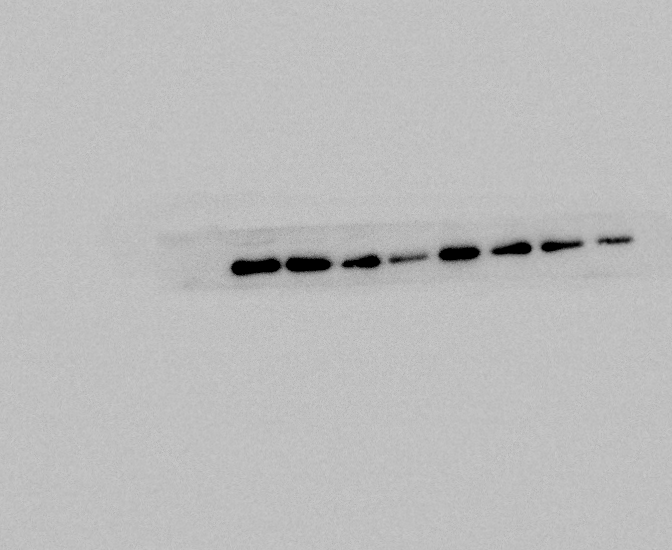
**

**KG-1α**: GAPDH **
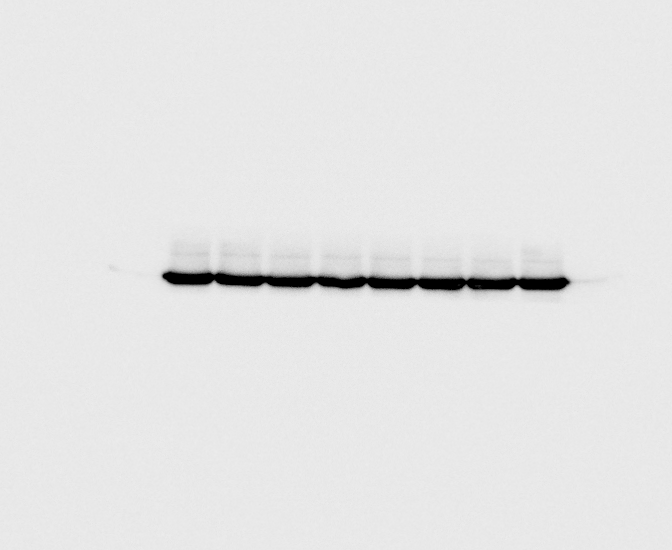
**

**Kasumi-1**: p-PI3K
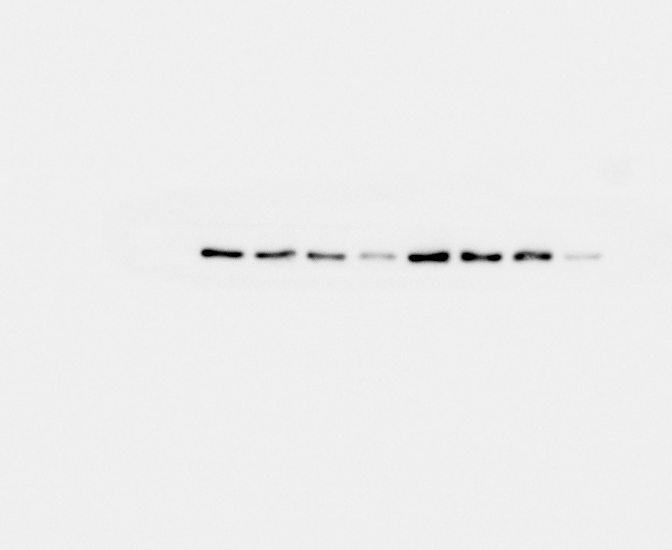


**Kasumi-1**: GAPDH **
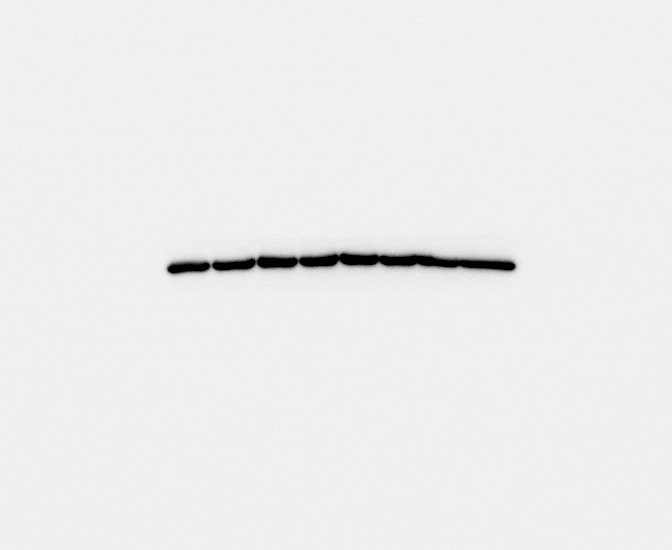
**

**Fig. 4D**

**KG-1α**: p-PI3K
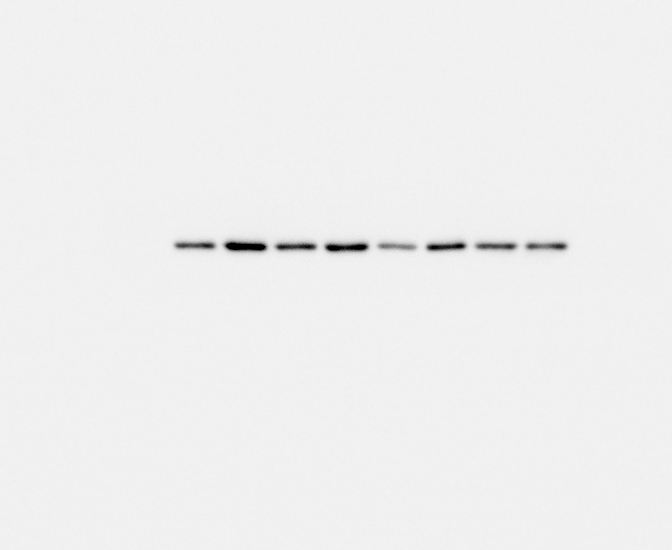


**KG-1α**: GAPDH
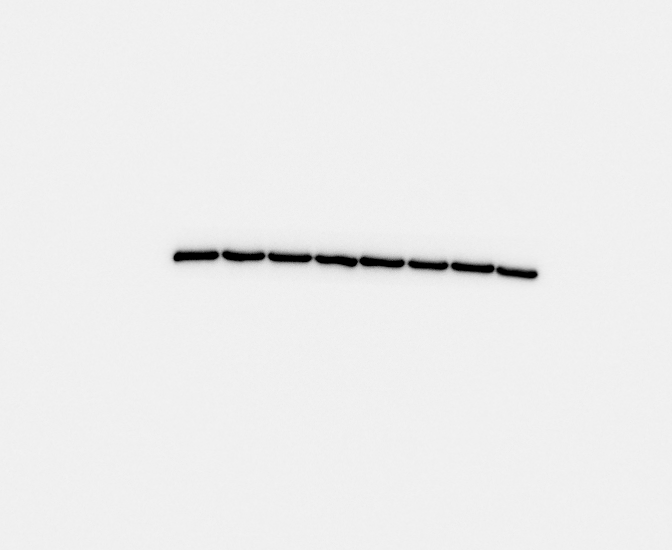


**Kasumi-1**: p-PI3K
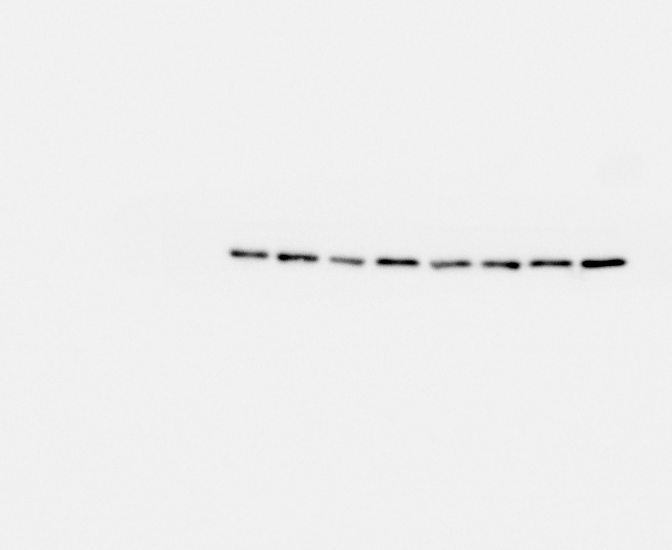


**Kasumi-1**: GAPDH
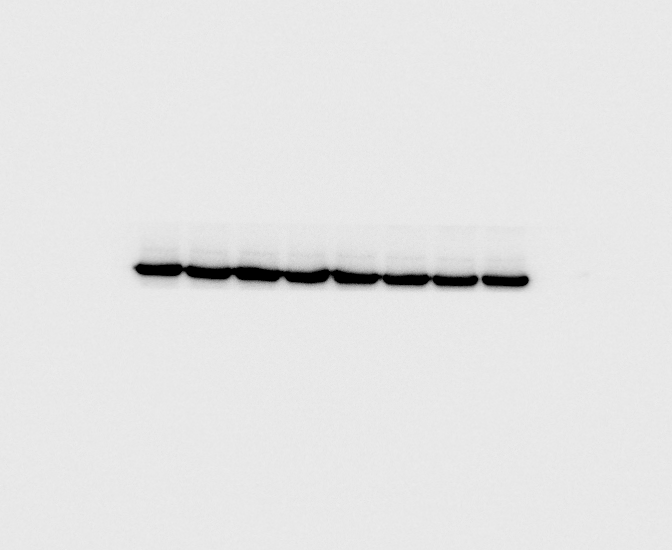


**Fig. 4F**

PPARα
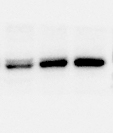
 PPARα
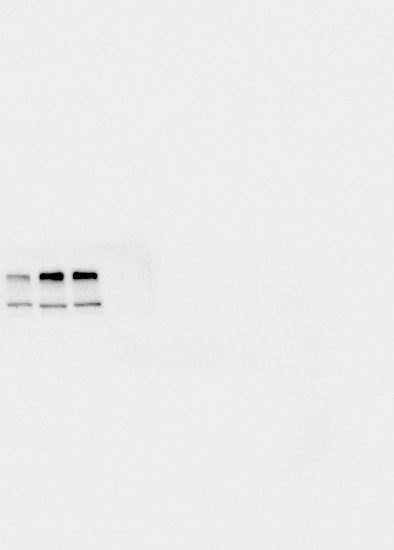


p-PI3K
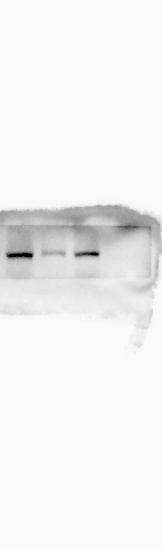
 p-PI3K
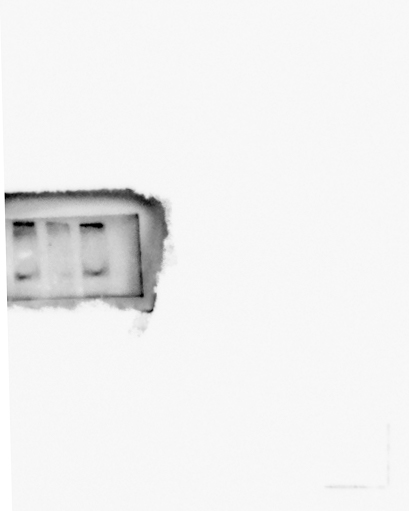


p-Akt1
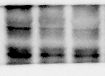
 p-Akt1
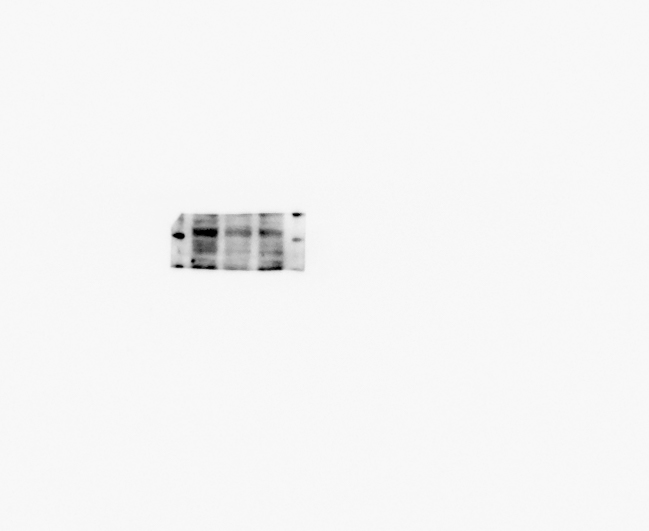


Bcl-2
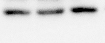
 Bcl-2
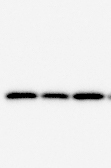


Bcl-xL
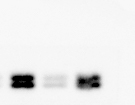
 Bcl-xL
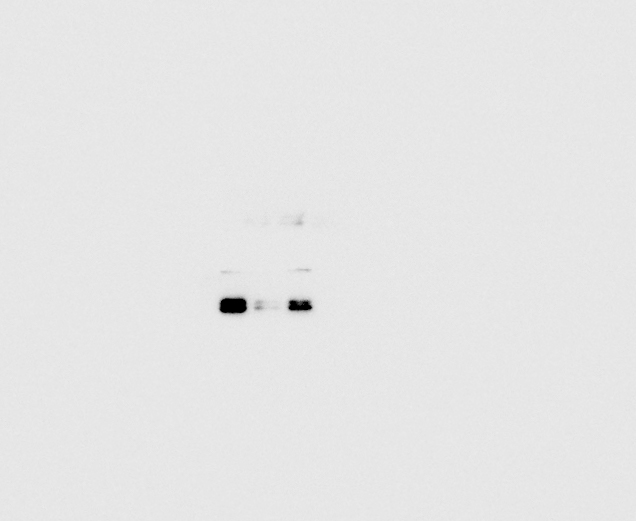


Mcl-1
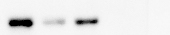
 Mcl-1
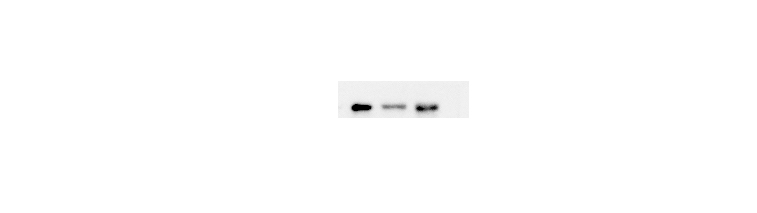


Bim
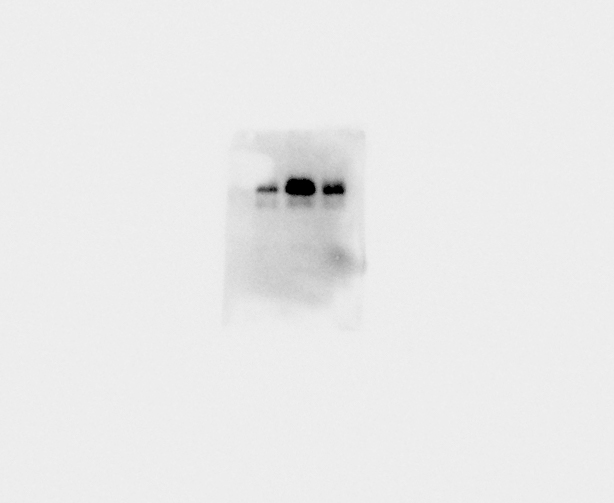
 Bim
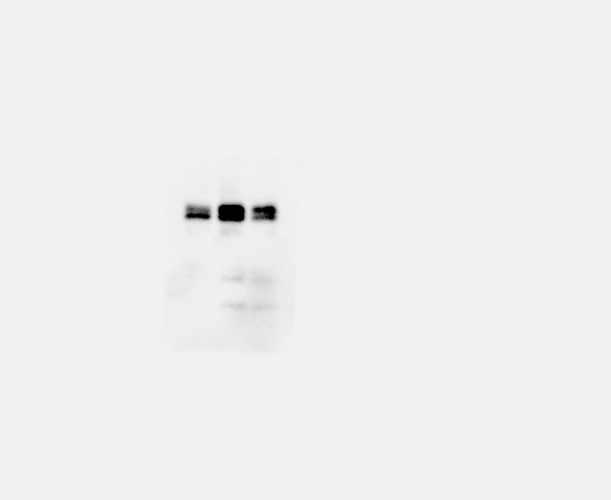


GAPDH
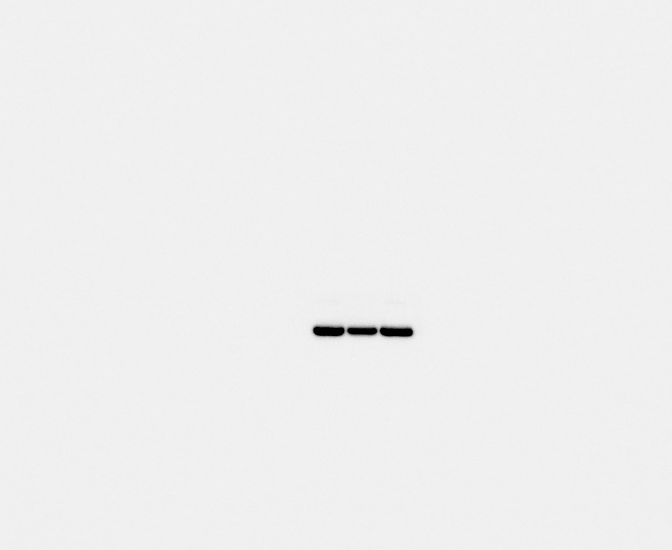
 GAPDH
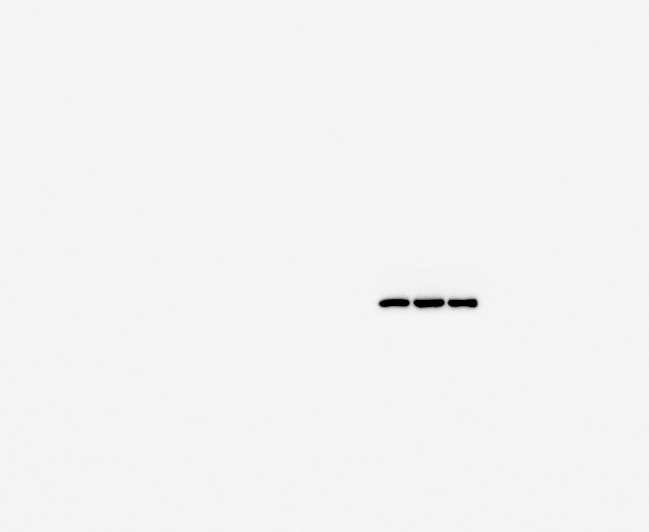


**KG-1α Kasumi-1**

**Fig. 4H**

Bcl-2
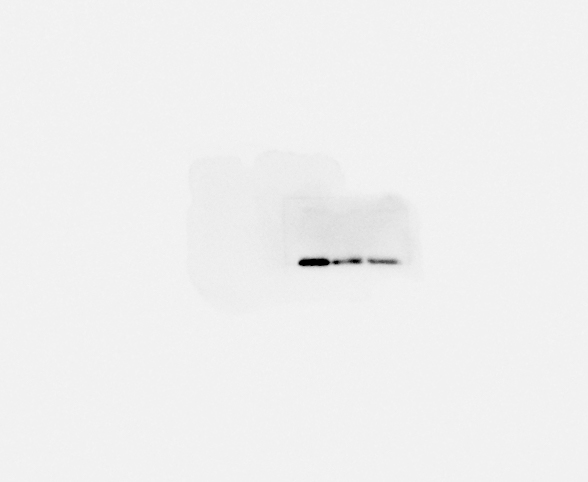
 Bcl-2
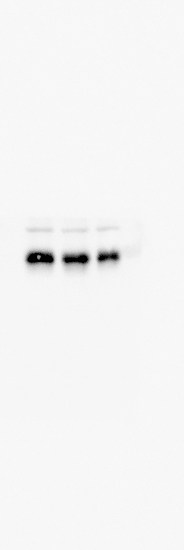


Bcl-xL
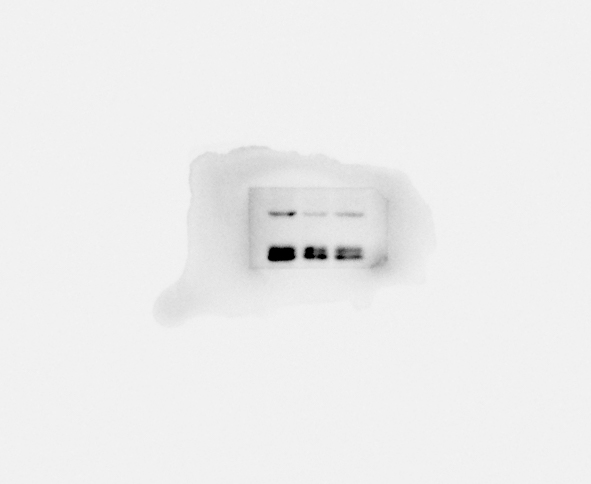
 Bcl-xL
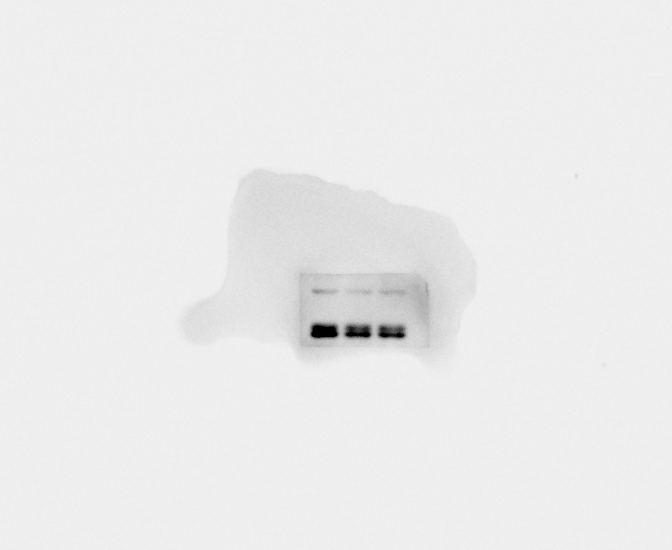


Mcl-1
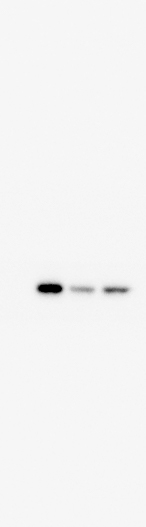
 Mcl-1
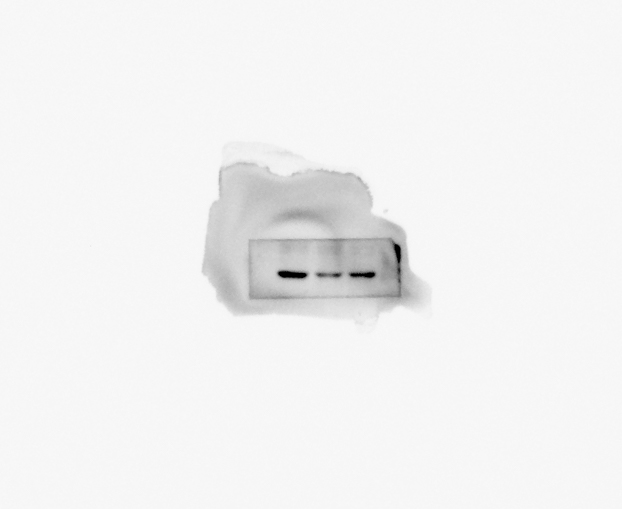


Bim
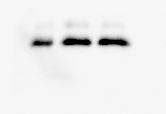
 Bim
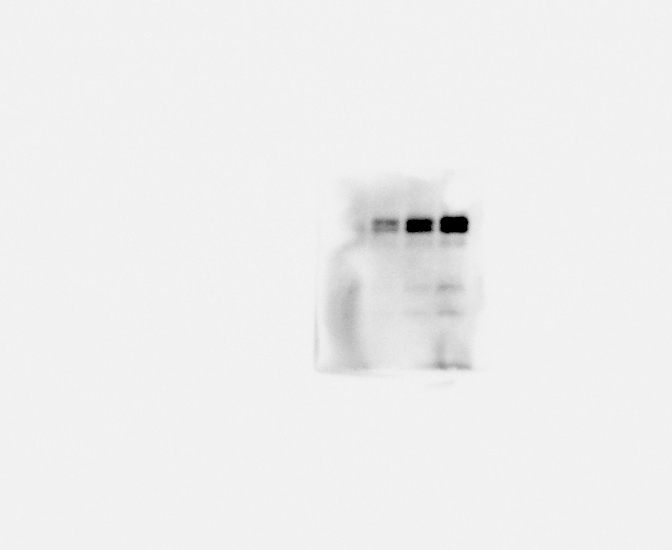


GAPDH
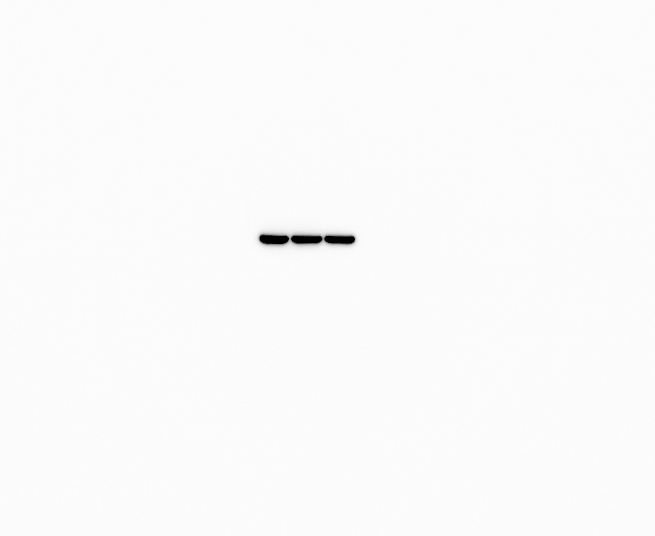
 GAPDH
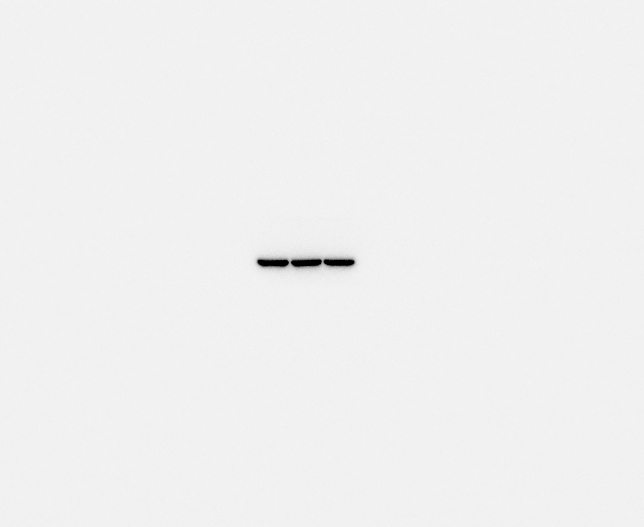


**KG-1α Kasumi-1**
